# Melatonin Improves Semen Quality by Modulating Oxidative Stress, Endocrine Hormones, and Tryptophan Metabolism of Hu Rams Under Summer Heat Stress and the Non-Reproductive Season

**DOI:** 10.3390/antiox14060630

**Published:** 2025-05-24

**Authors:** Qian-Qiu Liu, Xiong Li, Jia-Hao Li, Yang Zhou, Ming-Kai Lei, Wei-Qi Yin, You-She Ren, Chun-He Yang, Chun-Xiang Zhang

**Affiliations:** 1 College of Animal Science, Shanxi Agricultural University, Taigu 030801, China; 17749122998@163.com (Q.-Q.L.); 18335268252@163.com (X.L.); ljh_sxau8214@163.com (J.-H.L.); rys925@126.com (Y.-S.R.); 2 Shanxi Key Laboratory of Animal Genetics Resource Utilization and Breeding, Shanxi Agricultural University, Taigu 030801, China; zhouyang970428@163.com (Y.Z.); electorboy@163.com (M.-K.L.); yin18434762771@163.com (W.-Q.Y.)

**Keywords:** rams, heat stress, melatonin, sperm quality, tryptophan metabolites

## Abstract

The concurrent occurrence of summer heat stress and the non-breeding season has significantly impaired the semen quality of rams. Currently, there exists no straightforward and efficient method to address this issue. In this study, we demonstrate that two consecutive administrations of melatonin implants significantly mitigate the adverse effects of summer heat stress and the non-reproductive season on rams. Our findings indicate that implantation of exogenous melatonin enhances semen quality by improving sperm DNA integrity, mitochondrial integrity, and decreasing the proportion of abnormal sperm, as compared to control rams. This improvement is ascribed to the alleviation of oxidative stress and the optimized regulation of endocrine hormone levels in both serum and seminal plasma. Further exploration of the regulatory mechanism reveals that melatonin can also influence the tryptophan metabolism pathway. Additionally, our study revealed that certain indices and metabolites are strongly correlated with semen quality and can potentially serve as indicators for research aimed at improving semen quality. Notably, this is the first time that differences in tryptophan metabolites between serum and seminal plasma have been elucidated. All the above information suggests that melatonin implantation can protect sperm from heat stress by optimizing the blood and semen microenvironment.

## 1. Introduction

In the context of large-scale and intensive indoor sheep production, rams in China’s lamb meat industry are expected to carry out mating tasks year-round. However, the semen quality of most sheep breeds exhibits seasonal variations in regions with four distinct seasons situated between 23.5° N and 66.5° N or 23.5° S and 66.5° S latitude [1,2,3]. Particularly during summer, the semen quality of rams deteriorates due to acrosomal abnormalities [4] or morphological changes [5], which is exacerbated by lower antioxidant concentrations resulting from the dual effects of seasonal heat stress and the non-breeding season [6]. Moreover, heat stress also influences the secretion of endocrine hormones in several animal species, including reductions in reproductive hormones (LH, T, and FSH) [7,8,9], decreases in thyroid hormones (T3 and T4), and increases in cortisol levels in serum [10,11,12]. Therefore, it is crucial to implement efficient strategies to alleviate the adverse effects of heat stress on the semen quality of rams during the summer months.

Previous studies showed that the decline in semen quality may be attributed to reduced levels of the pineal hormone melatonin (N-Acetyl-5-methoxytryptamine) [13]. Numerous studies have demonstrated that melatonin possesses potent antioxidant properties [14,15], effectively scavenging reactive oxygen species to enhance rams’ sperm viability in vitro preservation [16,17,18]. Furthermore, several studies have demonstrated that melatonin can protect the epididymal epithelial cells from inflammation [19], alleviate oxidative stress in Leydig cells [20] in vitro, and thereby maintain the functions of the cells in the epididymis and testes. Additionally, melatonin implantation or administration has been employed to improve semen quality and sperm fertilization capacity by increasing testosterone secretion [21] and enhancing antioxidant systems [6,17], thereby promoting testicular development and reproductive function during the non-breeding season [22,23,24]. Other researchers have shown that subcutaneous implantation of slow-release melatonin has a protective effect on semen quality in various animals, including mithun [25], Beetal bucks [26], and ram [22].

However, the precise mechanisms by which melatonin enhances semen quality are yet to be fully elucidated. Melatonin is a tryptophan-derived metabolite in the tryptophan metabolism pathway [27]. Several studies reported that the addition of the optimal L-tryptophan in extenders could improve post-thaw progressive motility of buffalo (*Bubalus bubalis*) bull spermatozoa during cryopreservation in vitro [28]. Wang et al. found that the reduction in key metabolites, such as tryptophan and methionine, in human seminal plasma of azoospermia patients leads to a decrease in sperm quality [29]. Currently, limited research has been conducted to investigate whether melatonin enhances semen quality by modulating tryptophan metabolites in serum and seminal plasma. Based on the aforementioned studies, we hypothesize that melatonin may interact with other tryptophan metabolites to improve semen quality in rams during the summer non-breeding season.

Therefore, we selected Hu sheep, a Chinese breed capable of year-round semen collection but exhibiting reduced semen quality during summer months (June to September) [30], as the experimental animals. In this study, we examined the effects of slow-release melatonin implants on semen quality, oxidative stress, endocrine hormone levels, and tryptophan metabolites in both serum and seminal plasma of Hu rams. Furthermore, a correlation analysis was performed between the indices in serum, seminal plasma, and semen quality.

## 2. Materials and Methods

All experimental procedures and practices involving rams in this study were approved by the Animal Use and Care Committee of Shanxi Agricultural University, ensuring compliance with ethical guidelines for animal research. This study was assigned the approval number SXAU-EAW-2021G.IK.006011221.

### 2.1. Animals and Experimental Design

The experimental trial was carried out from 8 June to 12 September during the summer at the sheep farm of the College of Animal Science, Shanxi Agricultural University, China (37.2507° N, 112.3459° E). The climatic characteristics of Shanxi Province are predominantly characterized by a temperate monsoon climate with a distinct continental feature, due to its inland geographical position. During the experimental period, the daytime period increased to 14.75 h, and then gradually decreased to 13.07 h, including 13 d for the non-breeding season and 52 d for overlaps with the transitional period. The data on the duration of daylight were collected from official meteorological data.

Twenty healthy 3-year-old Hu sheep rams with a fleece length of 2 cm (*n* = 10), with a mean weight of 90 ± 3 kg and similar sperm motility (mean: 83.7% ± 5.5%), were randomly assigned to two groups and housed in four designated adjacent pens (5 rams per pen) within the same sheep barn with natural ventilation: the control group (CON) and the melatonin group (MT). The animals were fed a standardized total mixed ration (formulated according to NRC) [31] and were maintained under consistent management practices. Rams were fed twice daily at 7:00 and 19:00 and had ad libitum access to clean drinking water. The experimental period lasted 85 days, including a 10-day preparation phase and 75 days for the main experiment. During the 10-day preparation phase, firstly, experimental rams selected from different pens were allocated to four pens to facilitate their acclimatization to the new social environment; secondly, two wireless temperature and humidity recorders were installed for testing equipment and assessing the degree of heat stress; thirdly, two days before the start of the experimental period, all rams were weighed on two consecutive mornings prior to feeding to determine their accurate live weight. The dose of exogenous melatonin implants was set at 2 mg/kg body weight, as determined by our previous study in goats [32]. The precise number of pellets required for each ram was calculated and weighed. For the rams in the MT group, slow-release melatonin implants (purchased from Beijing Kangtai Institute of Biotechnology, Beijing, China) were subcutaneously administered behind the ear using a dedicated applicator gun on June 17 at midnight (from 23:00 to 24:00). In contrast, the rams in the CON group were subjected to a sham surgical procedure in accordance with the experimental protocol. Simultaneously, the initial blood sample was collected. The melatonin implants were released over 60 days. As a result, the implantation procedure was repeated on the 61st day of the trial period, as described in detail above.

### 2.2. Collection of Temperature and Humidity Data

The temperature and humidity data were recorded at 10 min intervals using a 4G wireless temperature and humidity recorder (TH40 G4-EX, Shenzhen Huahanwei, Shenzhen, China), which was suspended 1.5 m above the ground. Thereafter, the temperature and humidity index (THI) was calculated according to the following formula presented in Reference [33]:

THI = 0.8 Tr + [(RH/100) × (Tr − 14.4)] + 46.4

where Tr is the temperature (°C) and RH is the relative humidity (%).

The criteria for evaluating heat stress in rams were based on the findings reported by Li et al., who conducted their study on Dorper × Hu crossbred rams [34]: THI < 72 = no heat stress, 72 to < 79 = mild heat stress, 79 to < 88 = moderate heat stress, and over 88 = severe heat stress.

### 2.3. Collection of Blood and Semen Samples

Blood samples (10 mL per ram) were collected via the jugular vein between 23:00 and 24:00 on days 0, 45, 60, and 75 of the experiment. The samples were incubated in a water bath at 37 °C for 30 min and subsequently centrifuged at 3000× *g* for 10 min to isolate serum. The resulting serum was divided into aliquots (200 μL/aliquot), transferred into microcentrifuge tubes, and stored at −80 °C for subsequent analysis of serum antioxidant capacity parameters, endocrine hormones, and tryptophan metabolites.

Semen samples were collected on days 0, 45, 60, and 75 using an artificial vagina after morning feeding. Ejaculate volume (EV) was recorded, and a 100 μL aliquot was diluted at a ratio of 1:7 for sperm quality assessment (designated as the pre-diluted semen). The remainder of the sample was immediately centrifuged at 1000× *g* for 15 min. Subsequently, the seminal plasma was divided into aliquots and transferred into 100 μL microtubes for storage at −80 °C for subsequent analysis of antioxidants, endocrine hormones, and tryptophan metabolites.

### 2.4. Evaluation of Sperm Quality

In vitro sperm evaluations included the following parameters: Sperm motility (SM) was assessed using a computer-assisted semen analyzer, according to the method reported by Moses et al. [35]. Density (SD) was determined using a NucleoCounter SP-100™ (ChemoMetec company, Allerød, Denmark), after mixing of fresh semen (2.4 μL) and cell lysate (1 mL) in a 1.5 mL centrifuge tube. Subsequently, the pre-diluted semen was adjusted to a consistent concentration based on the sperm count and was named “the diluted semen”. Deformation rates were evaluated using the improved Giemsa staining method with the Quick Sperm Stain Kit (D029-1-1, Nanjing Jian cheng biotechnology Co., Ltd., Nanjing, China). Briefly, 8 μL of the diluted semen was evenly spread on a clean glass slide, followed by natural air-drying. The sample on the slide was fixed with 95% ethanol for 2–3 min. After complete drying, the sample was hydrated for 1–2 min. Subsequently, it was stained with the nuclear staining solution (Reagent I) for 45 s and rinsed thoroughly with running water. The cytoplasm staining solution (Reagent II) was then applied, followed immediately by a single rinse with the color-enhancing solution (Reagent III). The total count of morphologically abnormal spermatozoa was recorded on an optical microscope. Mitochondrial membrane integrity (MI) was measured by the JC/PI (HY-K0601 and HY-D0815, MedChemExpress Asia Agent, Shanghai, China) staining method [36]. DNA integrity (DNAI) was analyzed through the OA/EB double-staining method reported by Darzynkiewicz et al. (OA: Solebro Biotechnology Co., Ltd., Beijing, China; EB: Shanghai Aladdin Biochemical Technology Co., Ltd., Shanghai, China, respectively) [37]. Plasma membrane integrity of sperm (PMI) was determined by the hypo-osmotic swelling test as described by Alcay et al. [38]. Briefly, 20 μL of the diluted semen sample was added to each of the four 1.0 mL centrifuge tubes, followed by the sequential addition of 20 μL, 30 μL, 40 μL, and 110 μL of hypotonic solution (150 mOsm). After the samples were incubated at 37 °C for 30 min, the sperm tail curvature was examined using an optical microscope (Olympus DP72, Olympus Corporation, Tokyo, Japan). The above experiments were repeated three times. Fields of view were selected following a serpentine path to avoid overlapping imaging. The total number of sperm was no less than 400.

### 2.5. Determination of the Oxidative Stress Parameters and Hormone Levels

Determination of oxidative stress parameters: The activity of total superoxide dismutase (T-SOD) (A001-3-2) and catalase activity (CAT) (A007-1-1), as well as the levels of malondialdehyde (MDA) (A003-1), and the total antioxidant capacity (T-AOC) (A015-2-1) in serum and seminal plasma were measured using commercially available kits for serum and seminal plasma (Nanjing Jian cheng biotechnology Co., Ltd., Nanjing, China). Briefly, the principle of the T-SOD kit is based on the reaction system between xanthine and xanthine oxidase, which generates superoxide anion radicals. These radicals subsequently oxidize hydroxylamine to produce nitrite. The absorbance of the reaction mixture is measured at 550 nm in the presence of a chromogenic reagent using a visible-light spectrophotometer. When the sample contains superoxide dismutase (SOD), it specifically inhibits the generation of superoxide anion radicals, thereby reducing the formation of nitrite and resulting in a lower absorbance value in the test tube compared to the control. The SOD activity in the sample is then calculated using the provided formula. The determination of CAT activity is based on the principle that the decomposition of hydrogen peroxide (H_2_O_2_), catalyzed by catalase, can be rapidly terminated by the addition of ammonium molybdate. The residual H_2_O_2_ subsequently reacts with ammonium molybdate to form a yellow complex. The absorbance change in this complex can be quantified at 405 nm on the microplate reader, allowing for the calculation of CAT activity. The principle of MDA determination is that the MDA degradation products of peroxides can react with thiobarbituric acid to form a red-colored compound, which exhibits maximum absorbance at 532 nm. The absorbance was measured at 532 nm using a microplate reader, and the MDA concentration was subsequently calculated. The principle of T-AOC measurement is based on the ability of antioxidant substances to reduce Fe^3+^ ions to Fe^2+^ ions, which subsequently form stable complexes with phenanthroline derivatives. The degree of antioxidant capacity can subsequently be quantified at the wavelength of 520 nm. The T-AOC in the measured sample was calculated via the formula.

Measurement of hormone levels: The concentrations of sheep follicle-stimulating hormone (FSH) (H101-1-2), luteinizing hormone (LH) (H206-1-2), testosterone (T) (H090-1-1), triiodothyronine (T3) (H222-1-2), thyroxine (T4) (H223-1-2), and cortisol (COR) (H094-1-2) in serum and seminal plasma were quantified using commercial ELISA kits specially designed for sheep (Shanghai Enzyme Biotechnology Co., Ltd., Shanghai, China). These kits are developed based on the principle of a one-step double-antibody sandwich enzyme-linked immunosorbent assay (ELISA). Horseradish peroxidase (HRP)-conjugated detection antibodies specific to sheep are utilized, which bind to the seed hormones present in the samples. The chromogenic substrate 3,3′,5,5′-Tetramethylbenzidine is subsequently added and converted into a blue product by the enzymatic activity of peroxidase. Upon addition of an acidic stop solution, the color changes to yellow. A positive correlation exists between the intensity of the color and the concentration of the hormone in the sample, which can be quantified by measuring the absorbance at 450 nm using an RT6100 microplate reader from Rayto Life and Analytical Sciences Company (Shenzhen, China). The commercial ELISA kits exhibited a sensitivity of 0.1 mIU/mL for FSH, 1.0 pg/mL for LH and T, 1.0 ng/mL for COR, 0.1 nmoL/L for T3, and 1.0 nmoL/L for T4.

### 2.6. Targeted Tryptophan Metabolite Analysis

Tryptophan metabolites were quantified in 42 serum samples (collected on days 0, 45, and 60, *n* = 7) and 14 seminal plasma samples (collected on day 60, *n* = 7) using ultra-performance liquid chromatography coupled with tandem mass spectrometry (UHPLC-MS/MS). Briefly, a 100 μL aliquot of each sample was mixed with 400 μL of precooled extraction solution by vortexing for 30 s, followed by sonication for 5 min in an ice bath. The mixture was incubated at 40 °C for 1 h, and then centrifuged at 10,000× *g* at 4 °C for 15 min. A 400 μL aliquot of the supernatant was transferred to a new Eppendorf tube, dried under a gentle stream of nitrogen, and reconstituted in 100 μL of water containing 0.1% formic acid. After another centrifugation at 10,000× *g*, the clear supernatant was subjected to UHPLC-MS/MS analysis. The UHPLC separation was performed on an EXIONLC System (SCIEX company, Foster City, CA, USA) equipped with a Waters ACQUITY UPLC HSS T3 column (100 × 2.1 mm, 1.8 μm). Multiple reaction monitoring (MRM) data were acquired and processed using SCIEX Analyst Work Station Software (version 1.6.3) and SCIEX MultiQuant software (version 3.0.3). Thirty standards of tryptophan metabolites were purchased from Sigma-Aldrich Corporation, including L-tryptophan (Trp), L-5-Hydroxytryptophan (5-HTP), Serotonin (5-HT), N-Acetyl-5-hydroxytryptamine (NAS), and melatonin (MT); Kynurenine (KYN), Kynurenic acid (KYNA), 3-Hydroxyanthranilic acid (3-HAA), 3-Hydroxykynurenine (3-HK), 5-Hydroxyindoleacetic acid (5-HIAA), 5-Hydroxytryptophol (5-HTOL), 5-Methoxy-3-indoleacetic acid (5-Me-IAA), tryptamine (Try), Anthranilic acid (AA), Nicotinic acid (NiA), and Xanthurenic acid (Xa); and Indole, indican, Indole acrylic acid (IA), Indole-3-acetic acid (IAA), Indole-3-acetamide (IAM), Indole-3-carboxaldehyde (ICA), Indole ethanol/tryptophol (IE), 3-Indoleglyoxylic acid (IGA), Indolelactic acid (ILA), 3-Indolepropionic acid (IPA), Indoxylsulfate (IS), Indole-3-acetonitrile (IAN), Indole-3-acetyl-alanine (IAA-Ala), Indole-3-acetyl-aspartate (IAA-Asp), and skatole [39,40]. The lower limits of detection and lower limits of quantitation for the targeted tryptophan metabolites ranged from 0.01 to 195.31 nmol/L and 0.02 to 390.62 nmol/L, respectively. Correlation coefficients obtained from regression analysis were more than 0.9959 for all the analytes. The aforementioned testing work was commissioned to Shanghai Biotree Biotech Co., Ltd., Shanghai, China.

### 2.7. Statistical Analysis

The significant differences in semen quality, antioxidant indices, endocrine hormones, and melatonin were assessed using repeated measures analysis within the General Linear Mode (GLM) in SAS 9.4 (SAS Institute, Inc., Cary, NC, USA). The results were expressed as the mean ± standard error and plotted using GraphPad Prism 10.0. The levels of significance were defined as follows: *p* < 0.05 denotes a significant difference and *p* < 0.01 represents a highly significant difference. Heatmaps of tryptophan metabolites in serum and seminal plasma were drawn using R language version 3.6.3 (stats package and pheatmap package). Advanced correlation analyses linking semen quality indices with antioxidant capacity and endocrine hormone indices across animals at day 60 were conducted using R version 4.1.3, employing the psych package (2.4.1), ggplot2 package (3.3.3), and linkET package (0.0.7.4). The Mantel test was performed using the linkET package (version 0.0.7.4). The “r” values derived from the Mantel test, respectively, indicate negligible correlation when less than 0.4, moderate correlation when ranging from 0.4 to 0.6, and strong correlation when greater than or equal to 0.6. The "*p*" values derived from the Mantel test, respectively, indicate no significance when more than 0.05, a significant correlation when ranging from 0.05 to 0.01, and a marked significant correlation when less than 0.01. Heat map and correlation analyses were performed via an online platform available at https://www.omicstudio.cn/tool (accessed on 9 August 2024).

## 3. Results

This study demonstrates that the slow-release melatonin implantation mitigates oxidative stress, enhances the secretion of reproductive hormones, decreases cortisol secretion, and alters certain tryptophan metabolites in both serum and seminal plasma. Consequently, this improves the semen quality of Hu rams during both the summer season and the non-breeding season.

### 3.1. Heat Stress of Rams Housed During the Summer Season

**Figure 1:**
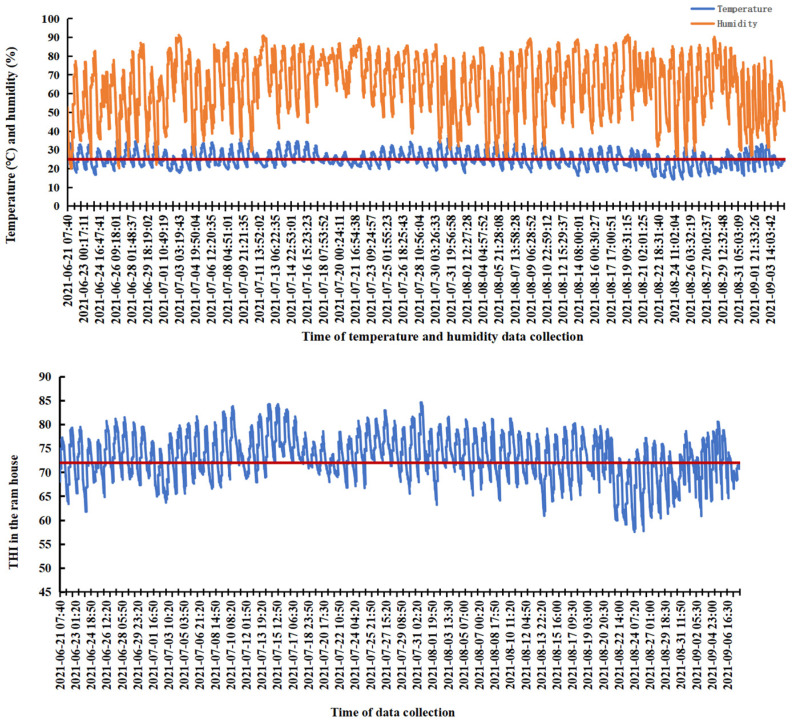
Variation curves of temperature, relative humidity, and the THI in the ram house.

The maximum, minimum, and mean values of temperature, relative humidity, and the THI in the ram house are summarized in the Supplementary Materials, Table S2. During the 75-day experimental period, the average temperature was recorded at 25.79 °C, with a peak value of 38 °C. The average relative humidity was measured at 65.04%, reaching a maximum of 91.40%. Consequently, the average THI was calculated to be 73.85, with a peak value of 84.63. These results indicate that the rams were subjected to varying degrees of heat stress throughout the experiment. Specifically, during the first 45 days (days 1 to 45), the highest recorded temperature was 38 °C (mean: 25.79 °C) and the highest THI reached 84.63 (mean: 74.13). From day 46 to day 60, the highest temperature decreased slightly to 34.50 °C (mean: 25.79 °C), and the highest THI dropped to 81.24 (mean: 72.92). From day 61 to day 75, the highest temperature further decreased to 33.10 °C (mean: 25.79 °C), and the highest THI was recorded at 80.61 (mean: 70.36). Figure 1 demonstrates that the longest continuous period of heat stress lasted for 114 h, extending from 07:02 am on 14 July to 11:14 pm on 18 July. Another prolonged period of heat stress occurred for 88 h, spanning from 07:25 am on 25 July to 01:23 am on 29 July. Throughout the experiment, the THI exceeded 72 on most days, indicating frequent exposure of the rams to heat stress conditions. Heat stress typically commenced around 07:00 am and persisted until approximately 02:00 am the following day. According to Figure 1, during the initial 45 days, both the intensity and duration of heat stress were relatively high, with the longest single-day heat stress lasting for 22 h. Between days 45 and 60, the duration and intensity of heat stress decreased significantly, accumulating only 15 h of moderate heat stress over 15 days. From day 60 to day 75, although moderate heat stress occurred for only 4 h in total, there were still approximately 7 h of mild heat stress per day, suggesting that the animals remained under some level of stress until the end of the experiment.

### 3.2. The Effect of Melatonin Implantation on the Semen Quality of Rams During the Summer Season

**Figure 2:**
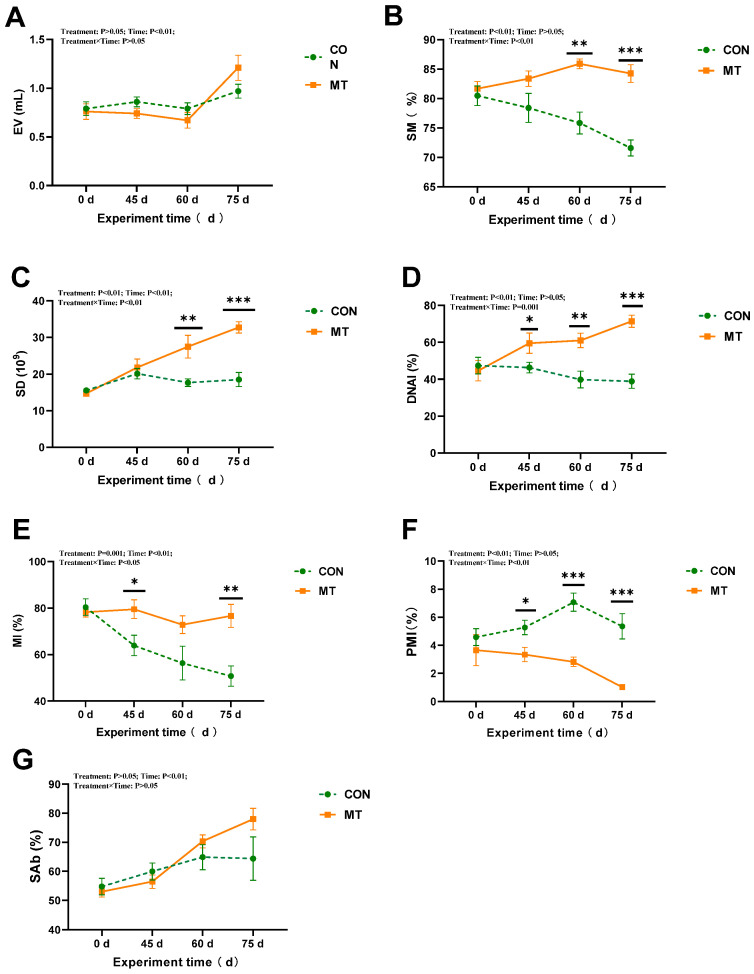
Impact of melatonin on semen quality of rams during the summer season (*n* = 10). (**A**) Ejaculate volume (EV). (**B**) Sperm motility (SM). (**C**) Sperm density (SD). (**D**) DNA integrity (DNAI). (**E**) Mitochondrial membrane integrity (MI). (**F**) Plasma membrane integrity (PMI). (**G**) Percentage of abnormal sperm (SAb). The solid yellow line indicates the MT group; the broken green line indicates the CON group. The data are expressed as mean ± SEM. The significant differences were assessed using repeated measures analysis. * *p* < 0.05, ** *p* < 0.01, and *** *p* < 0.001 between groups on that day.

Melatonin implantation significantly affected (*p* < 0.05) sperm motility, sperm density, sperm DNA integrity, sperm mitochondrial integrity, and the percentage of abnormal sperm during the experimental period from day 0 to day 75 (Figure 2B–F). Furthermore, a significant interaction was observed between melatonin implantation and duration of exposure. Specifically, for sperm motility and sperm DNA integrity, melatonin exhibited main effects as well as significant melatonin × days interactions (*p* < 0.01) (Figure 2B,D); moreover, with the extension of the implantation period, a significant enhancement in sperm motility and DNA integrity was observed in the MT group. For sperm density and mitochondrial integrity, there were significant effects of days (*p* < 0.01) and melatonin × days interactions (*p* < 0.05) (Figure 2C,E). As the experiment duration was extended, a significant decrease in the SD and MI was observed in the CON group, whereas a significant increase was noted in the MT group. Additionally, the MT group demonstrated a significantly lower percentage of abnormal sperm compared to the CON group (*p* < 0.01). No significant differences were observed in sperm plasma membrane integrity or ejaculate volume between the two groups (*p* > 0.05). However, significant time-dependent changes were noted in sperm plasma membrane integrity and ejaculate volume (Figure 2A,G), which increased as the experiment duration extended.

### 3.3. The Impact of Melatonin on Antioxidant Capacity in Serum and Seminal Plasma of Rams During the Summer Season

**Figure 3:**
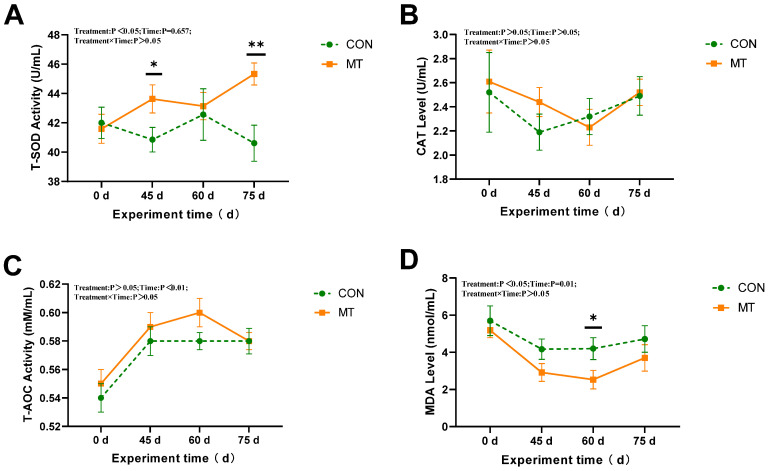
Impact of melatonin on antioxidant capacity parameters in the serum of rams (*n* = 10) during the summer season. (**A**) Total superoxide dismutase (T-SOD). (**B**) Catalase activity (CAT). (**C**) Total antioxidant capacity (T-AOC). (**D**) Malondialdehyde (MDA). The solid yellow line indicates the MT group; the broken green line indicates the CON group. The data are expressed as mean ± SEM. The significant differences were assessed using repeated measures analysis. * *p* < 0.05 and ** *p* < 0.01 between groups on that day.

Melatonin implantation significantly affected (*p* < 0.05) serum T-SOD activity and MDA content throughout the experimental period from day 0 to day 75 (Figure 3A,D). Compared with the CON group, the MT group exhibited a marked increase in serum T-SOD activity (*p* < 0.05) and a notable decrease in MDA content (*p* < 0.05). Days had a highly significant impact on T-AOC and MDA levels (*p* < 0.01) (Figure 3C,D). Specifically, on day 45, the T-SOD activity in the MT group was significantly elevated compared to that in the CON group (*p* < 0.05), whereas on day 60, the MDA content in the MT group was significantly lower than that in the CON group (*p* < 0.05). Melatonin implantation did not significantly affect CAT activity in serum (*p* > 0.05) (Figure 3B).

**Figure 4:**
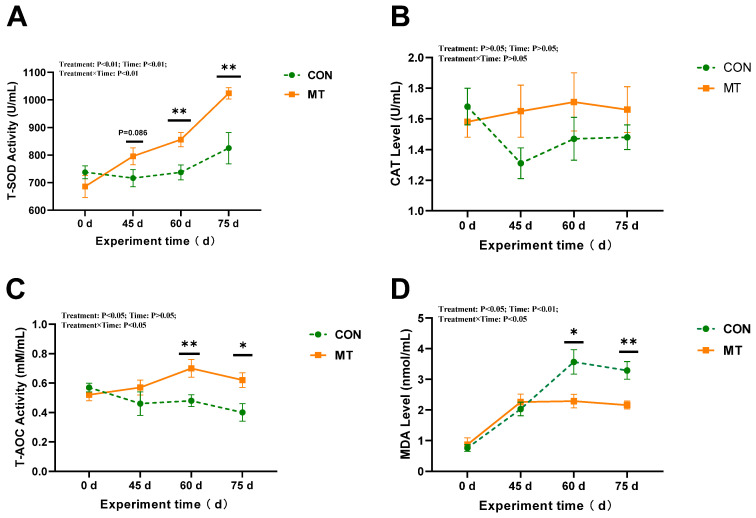
Impact of melatonin on antioxidant capacity parameters in seminal plasma of rams (*n* = 10) during the summer season. (**A**) Total superoxide dismutase (T-SOD). (**B**) Catalase activity (CAT). (**C**) Total antioxidant capacity (T-AOC). (**D**) Malondialdehyde (MDA). The solid yellow line indicates the MT group; the broken green line indicates the CON group. The data are expressed as mean ± SEM. The significant differences were assessed using repeated measures analysis. * *p* < 0.05 and ** *p* < 0.01 between groups on that day.

As shown in Figure 4, slow-release melatonin exerted a more pronounced effect on T-AOC and MDA levels (*p* < 0.05) (Figure 4C,D), as well as on T-SOD activity in seminal plasma (*p* < 0.01) during days 0 to 75 (Figure 4A). The T-SOD activity in seminal plasma of the MT group was significantly higher than that of the CON group on days 60 and 75 (*p* < 0.01) (Figure 4A). Additionally, T-AOC levels in the seminal plasma of the MT group were significantly elevated compared to those of the CON group on days 60 and 75 (*p* < 0.05) (Figure 4C). Significant day effects were observed for T-SOD activity and T-AOC (*p* < 0.01) and MDA content (*p* < 0.05) in seminal plasma, along with significant interactions between melatonin and days on T-SOD activity (*p* < 0.05). The T-SOD activity and T-AOC significantly increased with the extension of the experimental period in the MT group. However, with the increase in the experimental period, the MDA content exhibited a significant decrease in the MT group, whereas it demonstrated a significant increase in the control group. Melatonin implantation did not significantly influence CAT activity in seminal plasma (*p* > 0.05) (Figure 4B).

### 3.4. The Impact of Melatonin on Concentrations of Endocrine Hormones in the Serum and Seminal Plasma of Rams During the Summer Season

**Figure 5:**
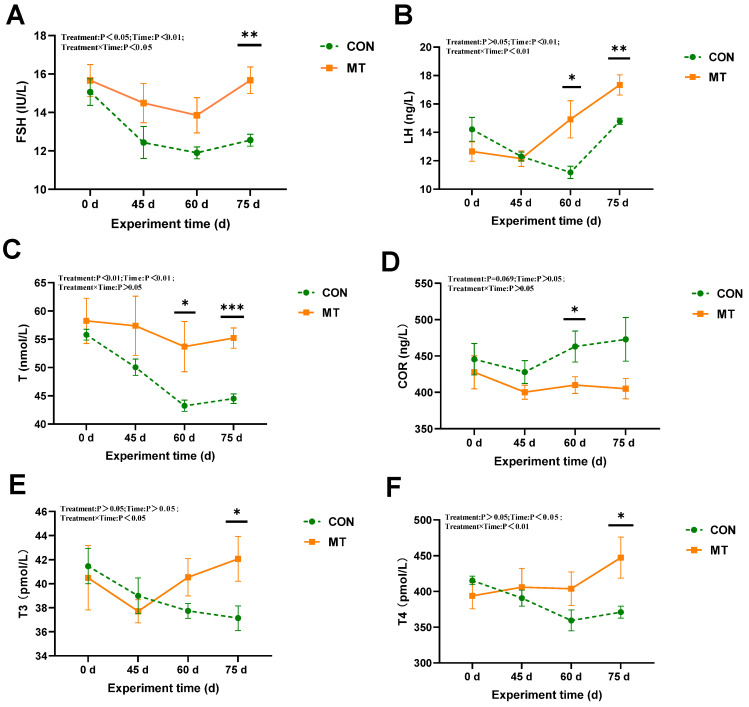
Impact of melatonin on endocrine hormones in the serum of rams (*n* = 10) during the summer season. (**A**) Follicle-stimulating hormone (FSH). (**B**) Luteinizing hormone (LH). (**C**) Testosterone (T). (**D**) Cortisol (COR). (**E**) Triiodothyronine (T3). (**F**) Thyroxine (T4). The solid yellow line indicates the MT group; the broken green line indicates the CON group. The data are expressed as mean ± SEM. The significant differences were assessed using repeated measures analysis. * *p* < 0.05, ** *p* < 0.01, and *** *p* < 0.001 between groups on that day.

The results demonstrated that slow-release melatonin significantly affected serum FSH and T3 concentration (*p* < 0.05) (Figure 5A,E), and exerted a highly significant impact on serum T concentration (*p* < 0.01) (Figure 5C). The cortisol concentration in the MT group was higher than that in the CON group at day 60 (*p* < 0.05) (Figure 5D). No significant effects were observed on other serum endocrine hormones (*p* > 0.05). Significant time-related effects were noted for serum concentrations of FSH, T, and LH (*p* < 0.01) (Figure 5A–C), as well as a significant time-related effect on T4 concentration (*p* < 0.05) (Figure 5F). The concentrations of FSH, LH, and T in the control group exhibited a significant decrease during days 0 to 60. Significant interactions between melatonin and treatment time were observed in the concentrations of LH and T4 (*p* < 0.01) (Figure 5B,F) and FSH and T3 (*p* < 0.05) (Figure 5A,E). By day 75, the MT group exhibited significantly elevated levels of FSH, LH, and T compared to the CON group (*p* < 0.01).

**Figure 6:**
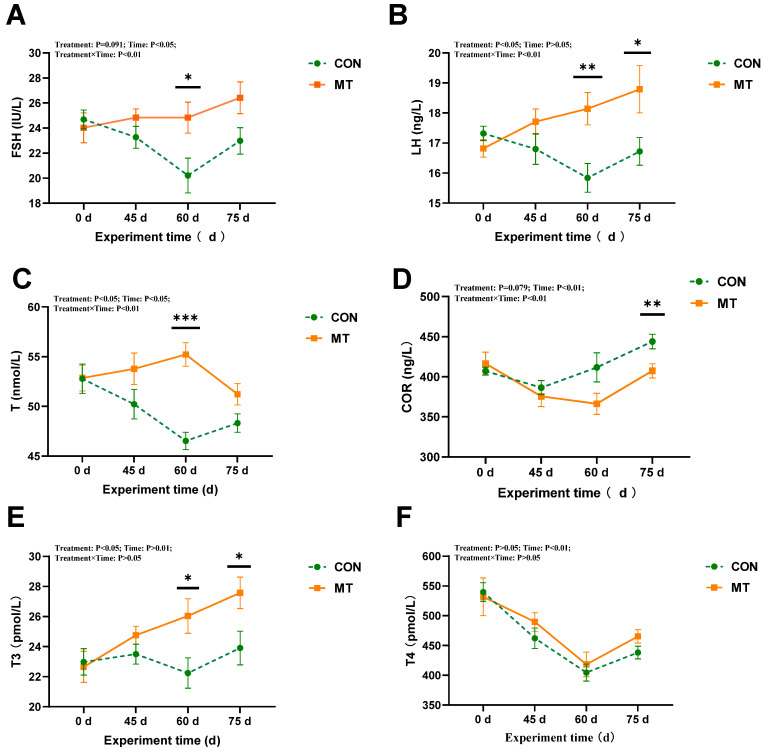
Impact of melatonin on endocrine hormones in seminal plasma of rams (*n* = 10) during the summer season. (**A**) Follicle-stimulating hormone (FSH). (**B**) Luteinizing hormone (LH). (**C**) Testosterone (T). (**D**) Cortisol (COR). (**E**) Triiodothyronine (T3). (**F**) Thyroxine (T4). The solid yellow line indicates the MT group; the broken green line indicates the CON group. The data are expressed as mean ± SEM, *n* = 10. The significant differences were assessed using repeated measures analysis. * *p* < 0.05, ** *p* < 0.01, and *** *p* < 0.001 between groups on that day.

The effects of slow-release melatonin on endocrine hormones in the seminal plasma of rams are presented in Figure 6. Slow-release melatonin significantly altered the concentrations of LH, T, and T3 in the seminal plasma (*p* < 0.05) (Figure 6B,C,E). Additionally, it induced a decreasing trend for cortisol (*p* = 0.079) (Figure 6D) and follicle-stimulating hormone (FSH) (*p* = 0.091) (Figure 6A). The MT group exhibited significantly higher T3 levels compared to the CON group at days 60 and 75 (*p* < 0.05) (Figure 6E). Furthermore, the duration of melatonin treatment had a significant effect on FSH (*p* < 0.05), T, cortisol, and T3 (*p* < 0.01). Specifically, FSH and T concentrations in the MT group were significantly elevated at day 60 (*p* < 0.05) compared to the CON group, while cortisol concentration was markedly reduced by day 75 (*p* < 0.01). Significant interactions between melatonin and treatment time were observed for FSH, LH, and T concentrations (*p* < 0.01) and cortisol concentrations (*p* < 0.05) in the seminal plasma (Figure 6A–D). Compared with the MT group, the concentrations of FSH and T significantly decreased (*p* < 0.05) and levels of LH numerically decreased in the CON group during the first 60 days of the experiment.

### 3.5. The Influence of Melatonin on the Key Tryptophan Metabolites Involved in Melatonin Biosynthesis in the Serum and Seminal Plasma of Rams

**Figure 7:**
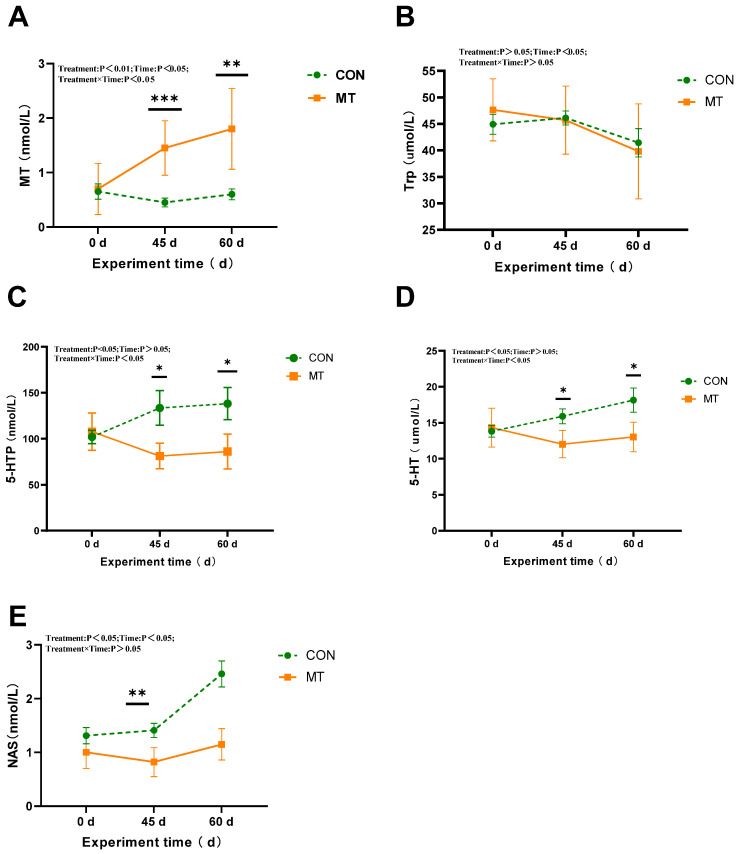
Impact of melatonin on the key tryptophan metabolites involved in melatonin biosynthesis in the serum of rams (*n* = 7) during the summer season. (**A**) Melatonin (MT). (**B**) L-tryptophan (Trp). (**C**) L-5-Hydroxytryptophan (5-HTP). (**D**) Serotonin (5-HT). (**E**) N-Acetyl-5-hydroxytryptamine (NAS). The solid yellow line indicates the MT group; the broken green line indicates the CON group. The data are expressed as mean ± SEM. The significant differences were assessed using repeated measures analysis. * *p* < 0.05, ** *p* < 0.01, and *** *p* < 0.001 between groups on that day.

The implantation of slow-release melatonin significantly influenced key tryptophan metabolites involved in melatonin biosynthesis in the serum (Figure 7). Specifically, slow-release melatonin led to a marked reduction in the concentrations of 5-HTP, 5-HT, and NAS (*p* < 0.05) (Figure 7C–E) while simultaneously causing a substantial increase in melatonin concentration (*p* < 0.01) in the serum during the period from day 0 to day 60 (Figure 7A). A significant day effect was observed for MT, Trp (Figure 7B), and NAS (*p* < 0.05). Additionally, significant interactions were observed between melatonin and day for MT, 5-HT, and 5-HTP (*p* < 0.05). The serum melatonin concentration began to increase significantly starting at day 45 (*p* < 0.01), with this effect persisting until day 60 of the test period (*p* < 0.05). However, as the experimental duration progressed, the concentrations of 5-HTP, 5-HT, and NAS in the CON group exhibited a marked increase on days 45 and 60 (*p* < 0.05).

**Figure 8:**
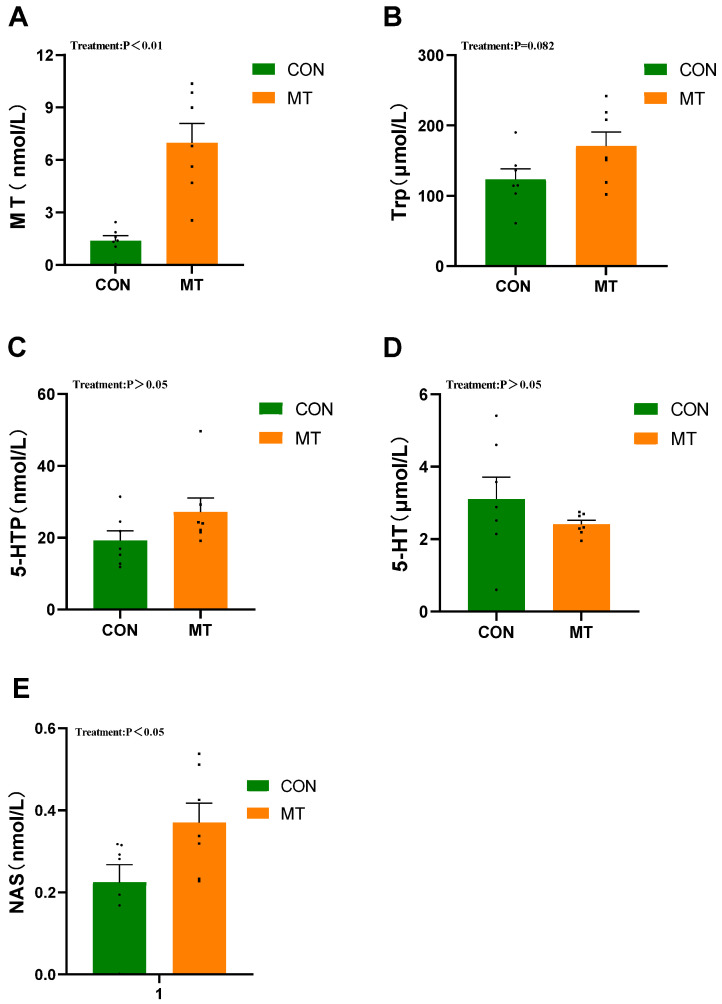
Impact of melatonin on the key tryptophan metabolites involved in melatonin biosynthesis in seminal plasma of rams (*n* = 7) during the summer season. (**A**) Melatonin (MT). (**B**) L-tryptophan (Trp). (**C**) L-5-Hydroxytryptophan (5-HTP). (**D**) Serotonin (5-HT). (**E**) N-Acetyl-5-hydroxytryptamine (NAS). The solid yellow line indicates the MT group; the broken green line indicates the CON group. The data are expressed as mean ± SEM.

Slow-release melatonin significantly increased the concentration of melatonin in seminal plasma (*p* < 0.01) (Figure 8A) and markedly elevated the levels of 5-HTP and NAS (*p* < 0.05) (Figure 8D,E) at day 60 compared with the CON group. Although the concentration of L-tryptophan in seminal plasma exhibited a decreasing trend, this change was not statistically significant (*p* = 0.081) (Figure 8B). Additionally, slow-release melatonin did not affect the concentration of 5-HT (*p* > 0.05) (Figure 8C).

**Figure 9:**
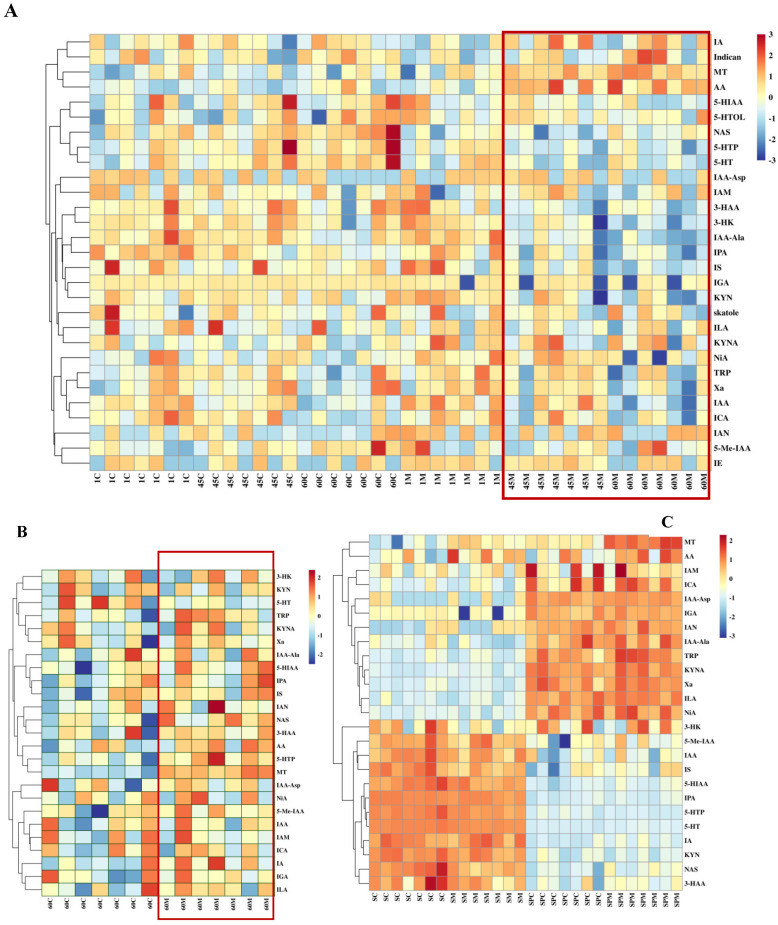
Heatmap of the key tryptophan metabolites in serum and seminal plasma of rams (*n* = 7). (**A**) Heatmap of the key tryptophan metabolites in serum at days 1, 45, and 60; (**B**) heatmap of the key tryptophan metabolites in seminal plasma at day 60; and (**C**) heatmap for comparison of common tryptophan metabolites in serum (SC and SM) and seminal plasma (SPC and SPM) at day 60. The labels 1 C, 45 C, and 60 C, and 1 M, 45 M, and 60 M represent the data of the control group and the treatment group at days 1, 45, and 60, respectively. SC, SM and SPC, SPM mean the control group and the treatment group in serum and seminal plasma at day 60, respectively. The red box section represents the treatment groups. All the abbreviations of tryptophan metabolites are presented as follows: L-tryptophan (Trp), L-5-Hydroxytryptophan (5-HTP), Serotonin (5-HT), N-Acetyl-5-hydroxytryptamine (NAS), Melatonin (MT), Kynurenine (KYN), Kynurenic acid (KYNA), 3-Hydroxyanthranilic acid (3-HAA), 3-Hydroxykynurenine (3-HK), Indole-3-acetic acid (IAA), 5-Hydroxyindoleacetic acid (5-HIAA), 5-Hydroxytryptophol (5-HTOL), Xanthurenic acid (Xa), 5-Methoxy-3-indoleacetic acid (5-Me-IAA), Anthranilic acid (AA), Nicotinic acid (NiA), Indole acrylic acid (IA), Indole-3-acetamide(IAM), Indole-3-carboxaldehyde (ICA), Indole ethanol/tryptophol (IE), 3-Indoleglyoxylic acid (IGA), Indolelactic acid (ILA), 3-Indolepropionic acid (IPA), Indoxylsulfate (IS), Indole-3-acetonitrile (IAN), Indole-3-acetyl-alanine (IAA-Ala), and Indole-3-acetyl-aspartate (IAA-Asp).

Targeted analysis of tryptophan metabolites identified a total of 29 tryptophan metabolites in the serum samples collected from rams (Figure 9A), with no detectable levels of tryptamine. Figure 9A showed that AA, MT, IA, and indican were clustered in a group. As shown in Figure 9B, only 25 were identified in the seminal plasma of rams, with no detectable levels of skatole, 5-HTOL, IE, and indican concentrations. Figure 9C illustrates the clustering of twenty-five common tryptophan metabolites shared between serum and seminal plasma, which are divided into four distinct areas. There were 11 tryptophan metabolites (including NAS, 5-HTP, and 50-HT) whose concentrations in serum are higher than those in seminal plasma, while 13 metabolites (including MT, Trp, AA, and ICA) have significantly higher concentrations in seminal plasma than in serum.

### 3.6. Correlation Analysis Between Semen Quality Parameters and Antioxidant Capacity and Endocrine Hormone Levels in the Serum and Seminal Plasma of Rams

**Figure 10:**
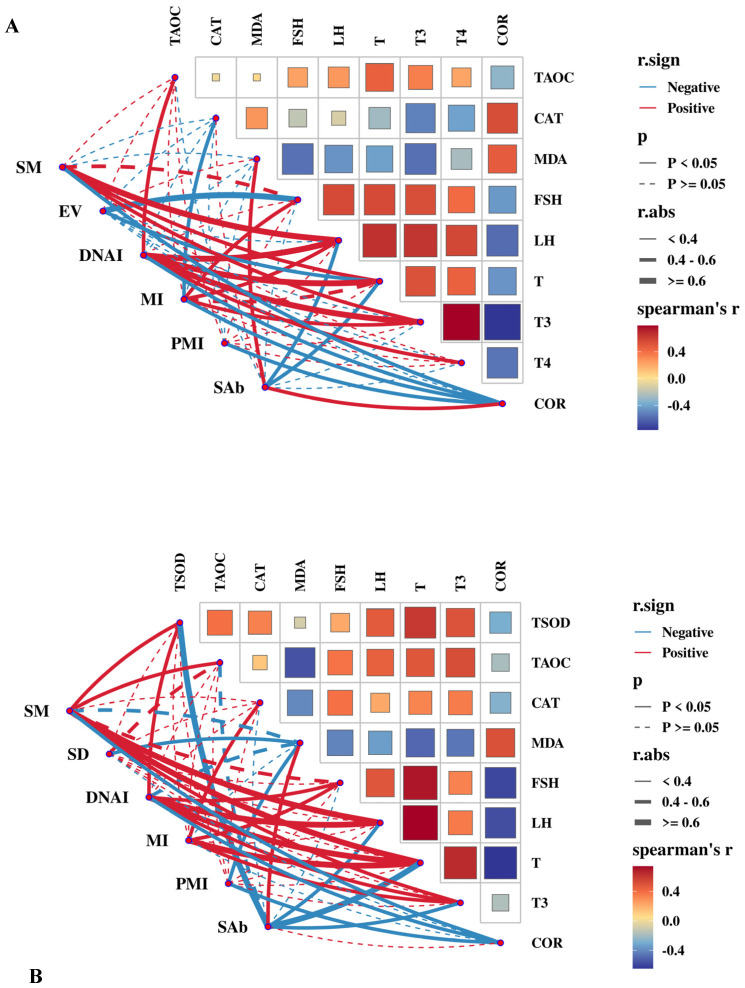
Color visualization of the correlation between semen quality parameters and the antioxidant capacities and endocrine hormones in serum (**A**) and seminal plasma (**B**). The spearman correlation matrix is visualized using colored boxes, where red denotes a positive association and blue indicates a negative association. The size of the boxes reflects the strength of the correlation. In the Mantel test, the colors of the connecting lines represent positive (red) and negative (blue) correlation coefficients. Solid and dashed lines denote statistically significant and non-significant correlations, respectively. The thickness of the connecting lines corresponds to the magnitude of the correlation coefficient, with bold lines representing a correlation coefficient of ≥0.6. All the abbreviations of tryptophan metabolites are presented as follows: sperm motility (SM), sperm density (SD), DNA integrity (DNAI), mitochondrial membrane integrity (MI), plasma membrane integrity (PMI), ejaculate volume (EV), total superoxide dismutase (T-SOD), catalase activity (CAT), total antioxidant capacity (T-AOC), malondialdehyde (MDA), follicle-stimulating hormone (FSH), luteinizing hormone (LH), testosterone (T), cortisol (COR), triiodothyronine (T3), and thyroxine (T4).

Advanced correlation between semen quality indices and serum antioxidants and endocrine hormones is shown in Figure 10A. Serum LH concentration was significantly positively strongly correlated with SM and DNAI (r > 0.6, *p* < 0.01), moderately positively correlated with MI (0.4 < r < 0.6, *p* < 0.05), and negatively correlated with SAb (0.4 < r < 0.6, *p* < 0.05). Serum T and T3 concentrations were significantly positively strongly correlated with DNAI (r > 0.6, *p* < 0.01) and moderately positively correlated with SM (0.4 < r < 0.6, *p* < 0.05). Serum FSH concentration was strongly negatively correlated with EV (r > 0.6, *p* < 0.01) and moderately positively correlated with MI (0.4 < r < 0.6, *p* < 0.05). Serum COR concentration was negatively correlated with SM, DNAI, and PMI, and positively correlated with SAb (0.4 < r < 0.6, *p* < 0.05).

Figure 10B shows that T-SOD activity in seminal plasma was positively moderately correlated with SM and DNAI (0.4 < r < 0.6, *p* < 0.05) and negatively strongly correlated with SAb (r > 0.6, *p* < 0.01). T-AOC in seminal plasma was positively correlated with SM (0.4 < r < 0.6, *p* < 0.05). T concentration (r > 0.6, *p* < 0.01) and T3 concentration (0.4 < r < 0.6, *p* < 0.05) in seminal plasma were significantly positively correlated with SM, DNAI, and MI and significantly negatively correlated with SAb. The LH concentration in seminal plasma was also significantly positively strongly correlated with SM and DNAI (r > 0.6, *p* < 0.01) and negatively correlated with SAb (0.4 < r < 0.6, *p* < 0.05). The MDA concentration was positively correlated with SAb (0.4 < r < 0.6, *p* < 0.05) and negatively correlated with SD and PMI (0.4 < r < 0.6, *p* < 0.05). The COR concentration in seminal plasma was moderately negatively correlated with SM, DNAI, and PMI (0.4 < r < 0.6, *p* < 0.05).

### 3.7. Correlation Analysis Between Semen Quality Parameters and Tryptophan Metabolites in the Serum or Seminal Plasma of Rams

**Figure 11:**
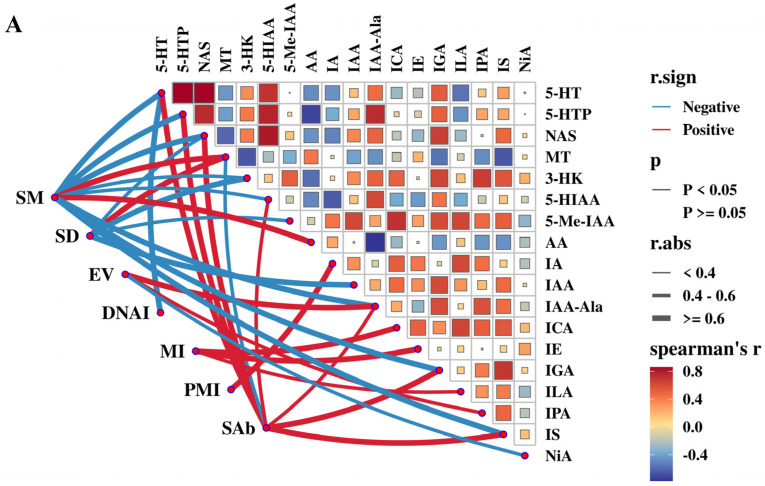

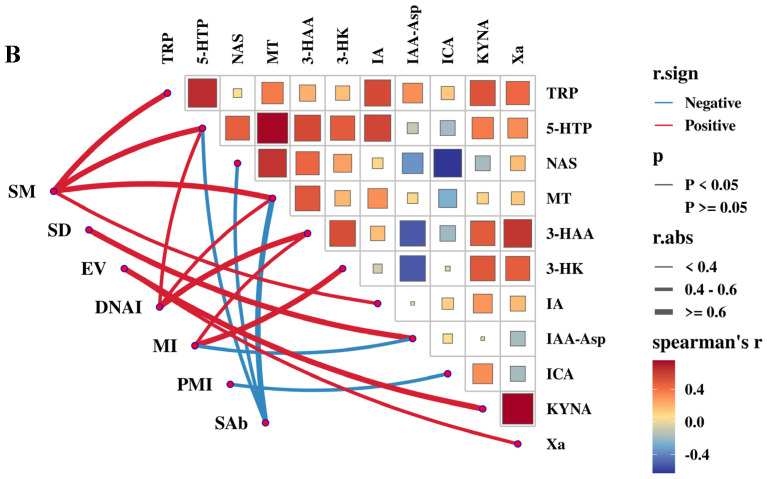
Color visualization of the correlation between semen quality parameters and tryptophan metabolites in serum (**A**) and seminal plasma (**B**). The spearman correlation matrix is visualized using colored boxes, where red denotes a positive association and blue indicates a negative association. The size of the boxes reflects the strength of the correlation. In the Mantel test, the colors of the connecting lines represent positive (red) and negative (blue) correlation coefficients. Solid and blank lines denote statistically significant and non-significant correlations, respectively. The thickness of the connecting lines corresponds to the magnitude of the correlation coefficient, with bold lines representing a correlation coefficient of ≥ 0.6. All the abbreviations of tryptophan metabolites are presented as follows: sperm motility (SM), sperm density (SD), DNA integrity (DNAI), mitochondrial membrane integrity (MI), plasma membrane integrity (PMI), ejaculate volume (EV), sperm motility (SM), L-tryptophan (Trp), L-5-Hydroxytryptophan (5-HTP), Serotonin (5-HT) N-Acetyl-5-hydroxytryptamine (NAS), Melatonin (MT), Kynurenine (KYN), Kynurenic acid (KYNA), 3-Hydroxyanthranilic acid (3-HAA), 3-Hydroxykynurenine (3-HK), 5-Hydroxyindoleacetic acid (5-HIAA), 5-Hydroxytryptophol (5-HTOL), 5-Methoxy-3-indoleacetic acid (5-Me-IAA), Anthranilic acid (AA), Nicotinic acid (NiA), Xanthurenic acid (Xa), Indole acrylic acid (IA), Indole-3-acetic acid (IAA), Indoxylsulfate (IS), Indole-3-acetamide(IAM), Indole-3-carboxaldehyde (ICA), Indole ethanol/tryptophol (IE), 3-Indoleglyoxylic acid (IGA), Indolelactic acid (ILA), 3-Indolepropionic acid (IPA), Indole-3-acetonitrile (IAN), Indole-3-acetyl-alanine (IAA-Ala), and Indole-3-acetyl-aspartate (IAA-Asp).

Figure 11A illustrates the advanced correlation between semen quality indices and serum tryptophan metabolites. The concentrations of 5-HTP, 5-HT, and NAS were significantly positively strongly correlated with SAb (r > 0.6, *p* < 0.01), while they were negatively strongly correlated with SM (r > 0.6, *p* < 0.01). MT concentration was significantly positively strongly correlated with both SM and SD (r > 0.6, *p* < 0.01). Serum Anthranilic acid (AA) concentration was significantly positively correlated with SM (r > 0.6, *p* < 0.01). Indole-3-carboxaldehyde (ICA) and Indole-3-ethanol (IE) concentrations were positively correlated with MI (r > 0.6, *p* < 0.01). The Indole-3-glycerol (IGA) and Indole-3-sulfonic acid (IS) concentrations were negatively correlated with SM and positively correlated with SAb (r > 0.6, *p* < 0.01). The Indole-3-acetic acid (IA) and IAA-Ala concentrations were significantly positively correlated with PMI and EV (r > 0.6, *p* < 0.01), respectively.

The advanced correlation link of semen quality indexes and tryptophan metabolites in the seminal plasma of rams is shown in Figure 11B. The concentrations of TRP, 5-HTP, and MT in seminal plasma were significantly positively strongly correlated with SM (r > 0.6, *p* < 0.01). The concentration of IAA-Asp was significantly positively correlated with SD (r > 0.6, *p* < 0.01). The concentrations of 3-Hydroxyanthranilic acid (3-HAA), 3-Hydroxykynurenine (3-HK), and Kynurenic acid (KYNA) were significantly positively strongly correlated with DNAI, MI, and EV (r > 0.6, *p* < 0.01), respectively. Additionally, MT concentration was negatively correlated with SAb (r > 0.6, *p* < 0.01).

## 4. Discussion

Modern large-scale indoor breeding of ewes has emerged as the predominant model for breeding and management practices for sheep production in China. Breeding rams are engaged in reproductive tasks year-round. Nevertheless, in northern China, where the seasons are well defined, high temperatures in summer can lead to a decline in semen quality of native rams in sheep production [31]. Several studies have reported rams are also subjected to heat stress when the THI exceeds 72 [7,41], leading to increased sperm abnormalities and decreased sperm motility [2,4,42]. In the present study, Hu rams were exposed to heat stress (THI > 73.85) for most of the day during the first 60 days of the experiment, which indicates that the rams were subjected to varying degrees of heat stress throughout the experiment. Heat stress did indeed lead to a decline in the semen quality of the Hu rams. The CON group exhibited decreased sperm motility, sperm density, DNA integrity, and mitochondrial integrity, as well as increased sperm abnormalities. The underlying cause of this decrease in semen quality can be attributed to two primary factors. In addition to heat stress induced by high temperatures, it is also influenced by the long duration of daytime, which places rams in the non-breeding season (early stage of the experiment) or the overlap between the non-breeding and breeding periods (the later stage of the experiment). Therefore, the Hu rams in our study suffered from both summer heat stress and non-reproductive period stress.

Melatonin, a potent antioxidant [14,15], can improve the testicular function in rams [22,43], thereby enhancing sperm motility [23,44]. Moreover, some studies indicated that melatonin implantation or administration has been shown to enhance fresh semen quality in Ossima rams [22] or Beetal bucks [26]. Moreover, melatonin plays a crucial role as a regulatory factor in mediating the photoperiodic response [45] and triggering seasonal reproduction [46]. Casao et al. reported that the concentration of melatonin and testosterone significantly decreases during the non-breeding season [13]. In this study, during the experimental period, the levels of FSH, LH, and T in the control group progressively decreased as the experiment progressed. The concentration of melatonin exhibited a numerical decline, but not to a statistically significant extent. These findings suggest that the rams remained in the mild anestrus phase throughout the experiment. Palacin et al. reported that the administration of melatonin implants to out-of-season rams 45 days before mating significantly enhances the fertilization capacity of ram spermatozoa and increases the lambing rate among mated ewes [47]. These findings suggest that melatonin plays a positive role in mitigating the heat stress-induced decline in semen quality and maintaining semen quality during non-breeding periods. In our study, the sperm DNA integrity and mitochondrial integrity of Hu rams significantly increased from the 45th day of MT implantation under the dual effect of non-breeding season and summer heat stress, which was in agreement with Shahat et al. [48], who found that melatonin administration before heat stress could improve sperm motility and morphology by 6 weeks. Because the duration for spermatogenesis and sperm transit through the epididymis is approximately 47 days in rams [49,50], melatonin implantation or injection should be conducted at least 45 days prior to the onset of heat stress to effectively safeguard rams against the adverse effects of heat stress and the non-breeding season.

In the current study, melatonin implantation was found to mitigate heat-induced oxidative stress by enhancing serum T-SOD activity and decreasing serum MDA content at day 60. These findings are consistent with those of Zhao et al. [44], who reported that melatonin could alleviate male infertility caused by heat-induced oxidative stress. Notably, in the MT group, T-SOD activity and T-AOC levels in seminal plasma were significantly elevated, while MDA was markedly reduced at both 60 and 75 days. These findings suggest that melatonin alleviated the oxidative damage in rams caused by heat stress during the summer season. Previous studies have shown that melatonin can enhance spermatozoa quality [16,51] by increasing T-AOC and reducing MDA levels, thereby mitigating oxidative stress induced by low-temperature preservation in vitro [17,52]. Another finding in this study is that the antioxidant system in seminal plasma is more sensitive to melatonin [13]. Especially, T-SOD is more sensitive. The activity of T-SOD in seminal plasma is extremely significantly higher than that in serum at 45 days. This suggests that melatonin promotes the enrichment of SOD in seminal plasma and enhances its activity. The regulatory mechanisms underlying this effect require further investigation.

Previous research has demonstrated that heat stress can influence the secretion of endocrine hormones in several animal species, including reductions in reproductive hormones (LH, T, and FSH) [7,8,9], decreases in the thyroid hormones (T3 and T4) [10], and increases in serum cortisol levels [11,12]. However, some studies have reported no significant effects of heat stress on LH concentration [53], T concentration [54], FSH level [41], COR [55], and T3 and T4 [56]. These discrepancies may be attributed to the differences among the experimental animals, variations in heat stress protocols, intensity, and duration of the exposure, as well as the variations in blood collection time points. Based on the circadian rhythm of melatonin secretion, we chose to collect blood samples between 23:00 and 24:00 at night, which corresponds to the peak period of melatonin secretion [57,58]. In the present study, the melatonin implantation significantly enhanced serum and seminal plasma concentrations of FSH, LH, and T at days 60 and 75, serum levels of T3 and T4 at day 75, and seminal plasma T3 level at day 60, while reducing serum and seminal plasma cortisol levels at days 60 and 75. Serum T concentration showed an increase at day 45, which is consistent with findings by Pool et al. [14] and Kokolis et al. [24], who observed serum T concentration increase at 6 weeks and 45 days post-implantation, respectively. Previous studies have confirmed that stress-induced elevations of cortisol can suppress testosterone secretion in bulls [59,60] and men [61]. Melatonin implantation decreased cortisol concentration and increased FSH, LH, and T concentrations, thereby enhancing the heat tolerance of animals [9]. This suggests that melatonin implantation mitigates the adverse effects of heat stress and the non-breeding season by regulating endocrine hormone secretion.

Furthermore, the secretion of melatonin exhibits a distinct circadian rhythm [62,63]. This circadian rhythm remains consistent across different seasons, characterized by a consistently similar baseline secretion level, while variations are specifically observed in the nighttime peak levels [64]. Zaidan et al. found that exogenous melatonin increases the amplitude of the endogenous melatonin peak in humans [65]. Further research by Bothorel demonstrated that the suprachiasmatic nucleus serves as the target site through which exogenous melatonin modulates the amplitude of the endogenous melatonin rhythm [66]. The study also observed a significant increase in the peak amplitude of melatonin following subcutaneous melatonin injection in rats. Moreover, the secretion of LH and T in rams occurred at regular intervals, exhibiting a similar fluctuation pattern (3~5 times) within a 24 h period [67]. The onset of LH release was followed by a sudden and dramatic increase in serum T levels. However, the number of LH and T pulses is different in winter and autumn, with the lowest number in winter. Some studies have shown that exogenous melatonin does not alter the 24 h rhythm of LH and T in humans [68], while other studies suggest that exogenous melatonin can influence the spontaneous release of LH in humans [69]. Therefore, in the subsequent phase of research, it is essential to further examine the effects of melatonin implantation on the endogenous melatonin secretion rhythm and the 24 h rhythms of LH and T, as well as their underlying molecular mechanisms.

Melatonin, also known as N-Acetyl-5-methoxytryptamine, is synthesized from tryptophan through a series of enzymatic reactions including hydroxylation, decarboxylation, acetylation, and methylation [70]. Several studies have reported that melatonin implantation results in elevated serum melatonin concentration in rams at 6 weeks, and increased levels in seminal plasma from 1 to 8 weeks during the non-breeding season [22]. We wondered whether the elevation in melatonin levels may influence the balance of tryptophan and its metabolites. To further investigate this, we conducted a targeted analysis of the tryptophan metabolism in serum at days 1, 45, and 60, and seminal plasma at day 60 using UHPLC-MS/MS methods [38,39]. In this study, a comprehensive analysis identified a total of 29 tryptophan metabolites in serum. In seminal plasma, 25 out of these metabolites were detected, with the exception of skatole, 5-Hydroxytryptophol, Indole-3-ethanol, and indican. This suggests that certain tryptophan metabolites present in the blood may not penetrate the blood–testis barrier and the blood–epididymal barrier to enter the testis and epididymis. The melatonin implantation altered some tryptophan metabolites during summer heat stress. There is little study about the impact of melatonin on tryptophan metabolism [71]. We found the serum melatonin concentrations enhanced at days 45 and 60 post-treatment, while the concentrations of 5-HT, 5-HTP, and NAS reduced, which may be indicative of an accelerated endogenous melatonin synthesis process or accumulation [72]. In the seminal plasma of the MT group, the concentrations of TRP were significantly positively strongly correlated with SM, which is consistent with the reports in the existing literature [27,28]. Moreover, melatonin levels were more than two times higher than those in the CON group at day 60. Compared with melatonin levels in serum, melatonin levels in seminal plasma increased nearly twofold higher than in the CON group at day 60. This suggests that melatonin may be synthesized and accumulated in seminal plasma during the melatonin implantation because Gonzalez-Arto et al. confirmed that there are enzymes related to melatonin synthesis in the testes, epididymis, and accessory glands [73]. All these results indicate that the high level of melatonin in seminal plasma is partly derived from the blood and partly from the male reproductive organs. Furthermore, elevated melatonin can bind directly to MT1 and MT2 receptors on the surface of the sperm [20,74], and there is also a possibility that melatonin can non-specifically interact with sperm membrane to protect the mobility of the sperm [75], which still requires further in-depth research.

To further investigate the correlations between various seminal quality parameters, serum parameters, and seminal plasma indicators, and to provide more reliable technical indicators for future research, we conducted comprehensive correlation link analyses among these variables using the data at day 60. Both serum and seminal plasma cortisol levels can serve as negative indicators of SM, DNAI, and PMI under heat stress, whereas concentrations of LH, T, and T3 can act as positive indicators of SM, DNAI, and MI. Additionally, MT levels in serum and seminal plasma can serve as negative indicators of SAb and positive indicators of SM. Several other tryptophan metabolites in serum or seminal plasma were also associated with seminal quality parameters; however, these associations require further validation through further expansion of sample sizes and an increase in the number of collection time points. Alternatively, we propose establishing a dedicated heat stress model for rams using an environmental control chamber to facilitate a more detailed and in-depth investigation into the mechanisms by which melatonin influences tryptophan metabolites.

## 5. Conclusions

This study investigated the mechanism by which melatonin enhances the semen quality of heat-stressed Hu rams, with a focus on both serum and seminal plasma parameters. The results demonstrated that melatonin implantation enhanced antioxidant capacity, maintained the stability of endocrine hormones, and modulated tryptophan metabolism in the serum and seminal plasma, thereby optimizing the micro-components of serum and seminal plasma. Specifically, alterations in components of seminal plasma can improve the microenvironment for sperm survival. More importantly, the differences in tryptophan metabolites between serum and seminal plasma induced by exogenous melatonin were identified. These findings suggest that the tryptophan metabolic pathway plays a crucial role in enhancing semen quality in heat-stressed rams, a mechanism that has not been previously reported.

## Data Availability

For raw data, please contact the corresponding author.

## Supplementary Materials

The following supporting information can be downloaded at https://www.mdpi.com/article/10.3390/antiox14060630/s1. Table S1: the body weight of rams and dose of implantation in MT group; Table S2:Maximum, minimum and average values of temperature, relative humidity and temperature-Humidity Index (THI) in ram house; Table S3: The effect of melatonin on sperm quality of ram during the summer; Table S4: The effect of melatonin on serum antioxidant capacity of ram during the summer; Table S5: The effect of melatonin on antioxidant capacity in seminal plasma of ram during the summer; Table S6: The effect of melatonin on serum endocrine hormone of ram during the summer; Table S7: The effect of melatonin on seminal plasma endocrine hormone of ram during the summer; Table S8: Effects of exogenous melatonin on key tryptophan metabolites for melatonin biosynthesis in serum during the summer; Table S9: Effects of exogenous melatonin on key tryptophan metabolites for melatonin biosynthesis in seminal plasma during the summer. Table S10: The r values, *p* values, and rd, r.sign, and pd of Figure 10; Table S11: The r values, *p* values, and rd, r.sign, and pd of Figure 11.
