# Supplementary material for: Melatonin Improves Semen Quality by Modulating Oxidative Stress, Endocrine Hormones, and Tryptophan Metabolism of Hu Rams Under Summer Heat Stress and the Non-Reproductive Season
Source: Antioxidants (Basel). 2025 May 24;14(6):630. doi: 10.3390/antiox14060630 (PMC12189995; doi:10.3390/antiox14060630)
Supplement: Supplementary file 1 [file antioxidants-14-00630-s001.zip › Supplementary Table 3-9..pdf]

Supplementary Table 3-9.

Supplementary table 3 The effect of melatonin on sperm quality of ram during the summer

Items	Time	Control group	Treatment group	*P*-value		
				MT	Day	MT × Day
Ejaculate volume (mL)	1~75 d	0.85 ± 0.07	0.85 ± 0.11	0.885	0.003	0.171
	1 d	0.79 ± 0.07	0.76 ± 0.08	0.785		
	45 d	0.86 ± 0.05	0.74 ± 0.05	0.113		
	60 d	0.79 ± 0.06	0.67 ± 0.08	0.258		
	75 d	0.97 ± 0.07	1.21 ± 0.13	0.133		
Sperm motility (%)	1~75 d	76.83 ± 2.24	83.81 ± 1.19	<0.001	0.191	0.005
	1 d	80.48 ± 1.66	81.67 ± 1.21	0.570		
	45 d	78.41 ± 2.47	83.39 ± 1.31	0.092		
	60 d	75.77± 1.85^{A}	85.90 ± 0.83^{B}	0.002		
	75 d	71.60 ± 1.37^{A}	84.26 ± 1.50^{B}	<0.001		
Sperm density $\times 10^{9}$	1~75 d	17.94 ± 1.40	24.18 ± 2.94	<0.001	<0.001	<0.001
	1 d	15.50 ± 0.66	14.68 ± 0.66	0.393		
	45 d	20.10 ± 1.42	21.82 ± 2.25	0.528		
	60 d	17.66 ± 1.03^{A}	27.48 ± 3.09^{B}	0.007		
	75 d	18.48 ± 1.94^{A}	32.74 ± 1.55^{B}	<0.001		
DNA integrity of sperm (%)	1~75 d	43.08 ± 4.01	59.12 ± 5.42	<0.001	0.156	0.001
	1 d	47.38 ± 4.48	44.54 ± 5.48	0.693		
	45 d	46.31 ± 2.87^{b}	59.53 ± 5.50^{a}	0.047		
	60 d	39.79 ± 4.51^{B}	61.03 ± 3.94^{A}	0.002		
	75 d	38.84 ± 3.83^{B}	71.40 ± 3.29^{A}	<0.001		
Mitochondrial integrity of sperm (%)	1~75 d	62.83 ± 6.05	76.78 ± 3.78	0.001	0.002	0.020
	1 d	80.37 ± 3.57	78.20 ± 2.05	0.605		
	45 d	63.90 ± 4.36^{a}	79.48 ± 4.03^{b}	0.017		
	60 d	56.31 ± 7.24	72.81 ±3.81	0.059		
	75 d	50.74 ± 4.38^{A}	76.62 ± 4.95^{B}	0.001		
Plasma membrane integrity (%)	1~75 d	61.00 ± 4.77	64.45 ± 4.11	0.329	<0.001	0.432
	1 d	54.76 ± 2.83	52.99 ± 1.80	0.606		
	45 d	59.96 ± 2.95	56.45 ± 2.32	0.363		
	60 d	64.89 ± 4.31	70.35 ± 2.23	0.275		
	75 d	64.41 ± 7.48	77.99 ± 3.72	0.121		
Percentage of abnormal Sperm (%)	1~75 d	5.57 ± 0.82	2.71 ± 0.41	<0.001	0.265	0.004
	1 d	4.58 ± 0.60	3.65 ± 1.10	0.467		
	45 d	5.27 ± 0.52a	3.34 ± 0.51b	0.017		
	60 d	7.07 ± 0.65A	2.82 ± 0.33B	<0.001		
	75 d	5.35 ± 0.90A	1.02 ± 0.15B	<0.001		

Note:The results are shown as mean ± SEM. Data labelled a or b were compared and significant differences identified ($p < 0.05$)， A and B were compared and significant differences identified ($p < 0.01$). The same as follows.

Supplementary table 4 The effect of melatonin on serum antioxidant capacity of ram during the summer

Items	Time	Control group	Treatment group	*P*-value		
				MLT	Day	MLT× Day
SOD（U/mL）	1～75 d	41.51 ± 1.13^{b}	43.42 ± 0.97^{a}	0.044	0.657	0.080
	1 d	42.00 ± 1.06	41.59 ± 0.99	0.781		
	45 d	40.85 ± 0.84^{b}	43.63 ± 0.96^{a}	0.043		
	60 d	42.56 ± 1.76	43.14 ± 0.93	0.777		
	75 d	40.61 ± 1.23^{B}	45.33 ± 0.75^{A}	0.004		
CAT（U/mL）	1～75 d	2.38 ± 0.21	2.45 ± 0.17	0.626	0.497	0.823
	1 d	2.52 ± 0.33	2.61 ± 0.26	0.841		
	45 d	2.19 ± 0.15	2.44 ± 0.12	0.213		
	60 d	2.32 ± 0.15	2.23 ± 0.15	0.697		
	75 d	2.49 ± 0.16	2.52 ± 0.11	0.876		
T-AOC（mM/mL）	1～75 d	0.57 ± 0.01	0.58 ±0.01	0.298	<0.001	0.918
	1 d	0.54 ± 0.01	0.55 ± 0.01	0.562		
	45 d	0.58 ± 0.01	0.59 ± 0.01	0.311		
	60 d	0.58 ± 0.006	0.60 ± 0.01	0.248		
	75 d	0.58 ± 0.009	0.58 ± 0.006	0.664		
MDA（nmol/mL)	1～75 d	4.70 ± 0.68^{a}	3.59 ± 0.61^{b}	0.030	0.010	0.710
	1 d	5.70 ± 0.80	5.19 ± 0.40	0.577		
	45 d	4.17 ± 0.55	2.92 ± 0.48	0.108		
	60 d	4.20 ± 0.59^{a}	2.53 ± 0.50^{b}	0.044		
	75 d	4.72 ± 0.72	3.70 ± 0.71	0.329		

Supplementary table 5 The effect of melatonin on antioxidant capacity in seminal plasma of ram during the summer

Items	Time	Control group	Treatment group	*P-value*		
				MLT	Day	MLT × Day
SOD（U/mL）	1～75 d	754.05 ± 37.78	840.49 ± 48.66	0.008	<0.001	<0.001
	1 d	737.51 ± 23.34	686.17 ± 39.96	0.282		
	45 d	716.36 ± 30.91	795.62 ± 30.77	0.086		
	60 d	737.33 ± 26.61^{B}	856.14 ± 25.41^{A}	0.005		
	75 d	825.00 ± 56.54^{B}	1024.03 ± 20.75^{A}	0.004		
CAT（U/mL）	1～75 d	1.48 ± 0.12	1.65 ± 0.15	0.052	0.778	0.483
	1 d	1.68 ± 0.12	1.58 ± 0.10	0.556		
	45 d	1.31 ± 0.10	1.65 ± 0.17	0.094		
	60 d	1.47 ± 0.14	1.71 ± 0.19	0.331		
	75 d	1.48 ± 0.08	1.66 ± 0.15	0.327		
T-AOC（mM/mL）	1～75 d	0.48 ± 0.06	0.60 ± 0.05	0.036	0.301	0.012
	1 d	0.57 ± 0.03	0.52 ± 0.04	0.387		
	45 d	0.46 ± 0.08	0.57 ± 0.05	0.260		
	60 d	0.48 ± 0.04^{B}	0.70 ± 0.06^{A}	0.008		
	75 d	0.40 ± 0.06^{b}	0.62 ± 0.05^{a}	0.017		
MDA（nmol/mL)	1～75 d	2.41 ± 0.44	1.89 ± 0.28	0.015	<0.001	0.014
	1 d	0.76 ± 0.11	0.87 ± 0.22	0.659		
	45 d	2.03 ± 0.22	2.26 ± 0.26	0.507		
	60 d	3.57 ± 0.40^{a}	2.29 ± 0.22^{b}	0.013		
	75 d	3.29 ± 0.29^{A}	2.16 ± 0.13^{B}	0.003		

Supplementary table 6 The effect of melatonin on serum endocrine hormone of ram during the summer

Items	Time	Control group	Treatment group	MLT	Day	MLT× Day
	1~75 d	13.03 ± 0.69	15.26 ± 0.88	0.041		
	1 d	15.06 ± 0.69	15.67± 0.83	0.576		
	45 d	12.44 ± 0.83	14.49 ± 1.02	0.138	< 0.001	0.030
FSH （IU/L）	60 d	11.90 ± 0.31	13.86 ± 0.92	0.058		
	75 d	12.56 ± 0.31B	15.68 ± 0.69A	0.001		
	1~75	13.12 ± 0.67	14.26 ±1.06	0.177		
	1 d	14.21 ± 0.84	12.65 ± 0.69	0.168		
	45 d	12.30 ±0.31	12.15 ± 0.55	0.818	< 0.001	< 0.001
	60 d	11.18 ±0.43^{b}	14.92 ± 1.31^{a}	0.015		
LH （ng/L）	75 d	14.78 ± 0.22^{B}	17.34 ± 0.71^{A}	0.003		
	0~75	48.40 ± 1.91	56.13 ±3.95	< 0.001		
	1 d	55.81 ±0.95	58.25 ±3.99	0.558		
	45 d	50.06 ± 1.45	57.38 ±5.23	0.195	< 0.001	0.056
T （nmol/L）	60 d	43.24 ± 0.99b	53.70 ± 4.44a	0.034		
	75 d	44.50 ±0.85B	55.21 ± 1.81A	< 0.001		
	1~75 d	452.38 ± 22.54	410.76 ± 15.08	0.069		
	1 d	445.55 ±21.69	427.73 ± 22.74	0.578		
	45 d	427.98 ±15.73	400.19 ± 9.53	0.148	0.070	0.433
	60 d	463.06 ±21.23a	410.03 ± 11.57b	0.042		
COR （ng/L）	75 d	472.95 ±29.96	405. 11± 14.01	0.055		
	1~75 d	38.84 ±1.28	40.21 ± 1.86	0.442		
	1 d	41.47 ± 1.47	40.49 ± 2.67	0.752		
T_3 （pmol/L）	45 d	38.99 ± 1.49	37.71 ± 0.96	0.479	0.121	0.046
	60 d	37.74 ± 0.62	40.55 ± 1.56	0.112		
	75 d	37.14 ± 1.02b	42.07 ± 1.86a	0.032		
	1~75	384.19 ± 12.26	412.82 ± 24.46	0.243		
T_4 （pmol/L）	1 d	415.48 ± 5.87	393.89 ± 18.15	0.273		
	45 d	390.74 ± 11.23	406.00 ± 26.62	0.604	0.043	< 0.001
	60 d	359.46± 14.55	403.98 ± 23.37	0.123		
	75 d	371.07 ± 8.33b	447.41 ± 28.64a	0.020		

Suppl. table 7 The effect of melatonin on seminal plasma endocrine hormone of ram during the summer

Items	Time	Control group	Treatment group	*P*-value		
				MLT	Day	MLT × Day
FSH（IU/L）	1～75	22.79 ± 1.13	25.03 ± 1.11	0.091	0.035	0.002
	1 d	24.69 ± 0.75	24.02 ± 1.20	0.641		
	45 d	23.27 ± 0.88	24.84 ± 0.70	0.181		
	60 d	20.22 ± 1.39^{b}	24.84 ± 1.23^{a}	0.023		
	75 d	22.98 ± 1.05	26.42 ± 1.27	0.052		
LH（ng/L）	1～75	16.67 ± 0.45	17.86 ± 0.57	0.021	0.269	0.005
	1 d	17.32 ± 0.24	16.82 ± 0.29	0.201		
	45 d	16.80 ± 0.51	17.71 ± 0.42	0.187		
	60 d	15.84 ± 0.48^{B}	18.14 ± 0.54^{A}	0.005		
	75 d	16.72 ± 0.46^{b}	18.79 ± 0.79^{a}	0.036		
T（nmol/L）	1～75	49.46 ± 1.40	53.26 ± 1.33	0.022	0.002	0.000
	1 d	52.78 ± 1.48	52.85 ± 1.31	0.975		
	45 d	50.22 ± 1.49	53.77 ± 1.58	0.121		
	60 d	46.53 ± 0.87^{B}	55.22 ± 1.18^{A}	< 0.001		
	75 d	48.32 ± 0.92	51.22 ± 1.08	0.057		
COR（ng/L）	1～75	412.22 ± 12.69	391.42 ± 13.66	0.079	< 0.001	0.039
	1 d	407.05 ± 4.78	416.49 ± 14.03	0.532		
	45 d	386.41 ± 8.71	375.54 ± 12.82	0.492		
	60 d	411.58 ± 18.11	366.31 ± 13.18	0.058		
	75 d	443.84 ± 9.15^{A}	407.35 ± 8.81^{B}	0.010		
T_3（pmol/L）	1～75	23.24 ± 0.92	25.71 ± 1.11	0.028	0.077	0.206
	1 d	22.98 ± 0.88	22.65 ± 1.04	0.815		
	45 d	23.50 ± 0.66	24.76 ± 0.59	0.173		
	60 d	22.24 ± 1.01^{b}	26.04 ± 1.15^{a}	0.023		
	75 d	23.91 ± 1.12^{b}	27.58 ± 1.05^{a}	0.028		
T_4（pmol/L）	1～75	460.49 ± 20.95	476.28 ± 24.25	0.447	< 0.001	0.513
	1 d	539.83 ± 15.59	531.77 ± 31.66	0.818		
	45 d	462.10 ± 17.11	489.61± 15.52	0.249		
	60 d	404.41 ± 14.35	418.18 ± 20.67	0.591		
	75 d	438.23 ± 10.55	465.35 ± 11.30	0.097		

Suppl. table 8 Effects of exogenous melatonin on key tryptophan metabolites for melatonin biosynthesis in serum during the summer.

Items	Time	Control group	Treatment group	*P*-value		
				MT	Day	MT × Day
Melatonin (nmol/L)	1~60 d	0.57 ± 0.08	1.32 ± 0.32	<0.001	0.038	0.013
	1 d	0.65 ± 0.14	0.70 ± 0.47	0.871		
	45 d	0.45 ± 0.08^{A}	1.45 ± 0.50^{B}	<0.001		
	60 d	0.60 ± 0.10^{A}	1.80 ± 0.74^{B}	0.002		
L-Tryptophan (μmol/L)	1~60 d	44.17± 1.67	44.40 ± 5.33	0.937	0.016	0.542
	1 d	44.93± 1.89	47.66± 5.86	0.406		
	45 d	46.13 ± 1.33	45.72 ± 6.41	0.889		
	60 d	41.45 ± 2.68	39.82± 8.97	0.732		
L-5-Hydroxytryptophan (nmol/L)	1~60 d	124.57 ± 12.34	91.74 ± 1151	0.054	0.684	0.028
	1 d	101.96 ± 7.20	107.81 ± 20.27	0.620		
	45 d	133.51 ± 18.76^{a}	81.27 ± 13.77^{b}	0.042		
	60 d	138.25 ± 17.54^{a}	86.14 ± 19.04^{b}	0.034		
Serotonin (μmol/L)	1~60 d	15.95 ±0.97	13.13 ± 1.45	0.049	0.203	0.042
	1 d	13.81 ± 0.81	14.32 ± 2.70	0.722		
	45 d	15.90 ± 1.02^{a}	12.03 ± 1.89^{b}	0.018		
	60 d	18.15 ± 1.69^{a}	13.04 ± 2.05^{b}	0.036		
N-Acetyl-5-hydroxytryptamine (nmol/L)	1~60 d	1.73 ± 0.26	0.99 ± 0.20	0.039	0.038	0.156
	1 d	1.31± 0.15	1.00 ± 0.30	0.179		
	45 d	1.41± 0.13	0.82 ± 0.27	0.008		
	60 d	2.46 ±0.24^{a}	1.15± 0.29^{b}	0.076		

Suppl. Table 9 Effects of exogenous melatonin on key tryptophan metabolites for melatonin biosynthesis in seminal plasma during the summer

Items	Control group	Treatment group	P-value
L-Tryptophan (μmol/L)	123.29± 6.21	170.79 ± 16.73	0.082
L-5-Hydroxytryptophan (nmol/L)	3.10 ± 0.26	2.40 ± 0.09	0.141
Serotonin (μmol/L)	19.22 ± 4.60^{b}	25.72 ± 3.53^{a}	0.015
N-Acetyl-5-hydroxytryptamine(nmol/L)	0.22 ± 0.02^{b}	0.37 ± 0.03^{a}	0.042
Melatonin (nmol/L)	1.55 ± 0.12^{B}	6.98 ± 0.60^{A}	<0.001
