# Supplementary material for: Melatonin Improves Semen Quality by Modulating Oxidative Stress, Endocrine Hormones, and Tryptophan Metabolism of Hu Rams Under Summer Heat Stress and the Non-Reproductive Season
Source: Antioxidants (Basel). 2025 May 24;14(6):630. doi: 10.3390/antiox14060630 (PMC12189995; doi:10.3390/antiox14060630)
Supplement: Supplementary file 1 [file antioxidants-14-00630-s001.zip › Supplementary table S1.pdf]

Supplementary Table 1. the body weight of rams and dose of implantation in MT group

MT				CON	
**Number**	**Body weight (Kg)**	**MT dosage (mg)**	**Number of particles**	**Number**	**Body weight**
1	88.5	177	6	1	89
2	92	184	6	2	92
3	92.5	185	6.2	3	95.5
4	89.5	179	6	4	94
5	84.5	169	5.6	5	86
6	95	190	6.3	6	90
7	90	180	6	7	85
8	90.5	181	6	8	88.6
9	87.5	175	5.8	9	90.5
10	91.5	183	6	10	91.6
Average	90.15			Average	90.22
Note: The weight of melatonin particles is 30 mg/particle. Before embedding, weigh and embed according to the required dose.					
