# Supplementary material for: Melatonin Improves Semen Quality by Modulating Oxidative Stress, Endocrine Hormones, and Tryptophan Metabolism of Hu Rams Under Summer Heat Stress and the Non-Reproductive Season
Source: Antioxidants (Basel). 2025 May 24;14(6):630. doi: 10.3390/antiox14060630 (PMC12189995; doi:10.3390/antiox14060630)
Supplement: Supplementary file 1 [file antioxidants-14-00630-s001.zip › Supplmentary table S2.pdf]

Supplementary Table 2. Maximum, minimum and average values of temperature, relative humidity and temperature-Humidity Index (THI) in ram house

	**Temperature/℃**	**Relative humidity/%**	**THI**
	38.00 （MAX[1]）	91.30 （MAX）	84.63 （MAX）
	16.80 （MIN[2]）	20.00 （MIN）	61.80 （MIN）
1~45 d	26.03 （AVG[3]）	64.36 （AVG）	74.13 （AVG）
	34.50 （MAX）	91.40 （MAX）	81.24 （MAX）
	16.20 （MIN）	25.20 （MIN）	60.94 （MIN）
45~60 d	25.04 （AVG）	66.85 （AVG）	72.92 （AVG）
	33.10 （MAX）	90.30 （MAX）	80.61 （MAX）
	14.20 （MIN）	26.60 （MIN）	57.57 （MIN）
60~75 d	23.60 （AVG）	61.57 （AVG）	70.36 （AVG）

Note: THI:Temperature humidity index; MAX: maximum; MIN: minimum.

Maximum, minimum and average values of temperature, relative humidity and THI in ram house
