# Supplementary material for: Melatonin Improves Semen Quality by Modulating Oxidative Stress, Endocrine Hormones, and Tryptophan Metabolism of Hu Rams Under Summer Heat Stress and the Non-Reproductive Season
Source: Antioxidants (Basel). 2025 May 24;14(6):630. doi: 10.3390/antiox14060630 (PMC12189995; doi:10.3390/antiox14060630)
Supplement: Supplementary file 1 [file antioxidants-14-00630-s001.zip › Table S10The r values, p values, and rd, r.sign and pd of Figure 10.pdf]

Table S10. A: The r values, p values, and rd, r.sign and pd of Figure 10.A

sepc	env	r	p	rd	r.sign	pd
SM	TSOD	0.494736842	0.026578647	0.4 - 0.6	Positive	P < 0.05
SD	TSOD	0.322411118	0.165631257	< 0.4	Positive	P >= 0.05
EV	TSOD	0.1057103	0.657362824	< 0.4	Positive	P >= 0.05
DNAI	TSOD	0.533834586	0.015337047	0.4 - 0.6	Positive	P < 0.05
MI	TSOD	0.206039203	0.383475531	< 0.4	Positive	P >= 0.05
PMI	TSOD	0.153441154	0.518365255	< 0.4	Positive	P >= 0.05
SAb	TSOD	-0.611508129	0.004170831	>= 0.6	Negative	P < 0.05
SM	TAOC	0.572074132	0.008398278	0.4 - 0.6	Positive	P < 0.05
SD	TAOC	0.424962413	0.061792013	0.4 - 0.6	Positive	P >= 0.05
EV	TAOC	0.108113819	0.650043813	< 0.4	Positive	P >= 0.05
DNAI	TAOC	0.367331811	0.111100588	< 0.4	Positive	P >= 0.05
MI	TAOC	0.106913861	0.653694027	< 0.4	Positive	P >= 0.05
PMI	TAOC	0.230421752	0.328389942	< 0.4	Positive	P >= 0.05
SAb	TAOC	-0.439006149	0.052805851	0.4 - 0.6	Negative	P >= 0.05
SM	CAT	0.160270926	0.499684554	< 0.4	Positive	P >= 0.05
SD	CAT	0.139087914	0.5586667	< 0.4	Positive	P >= 0.05
EV	CAT	-0.180375535	0.446658279	< 0.4	Negative	P >= 0.05
DNAI	CAT	0.271632884	0.246657713	< 0.4	Positive	P >= 0.05
MI	CAT	0.562313744	0.009859016	0.4 - 0.6	Positive	P < 0.05
PMI	CAT	0.104629289	0.660664501	< 0.4	Positive	P >= 0.05
SAb	CAT	-0.278509617	0.234423667	< 0.4	Negative	P >= 0.05
SM	MDA	-0.437242381	0.0538761	0.4 - 0.6	Negative	P >= 0.05
SD	MDA	-0.446752393	0.048296381	0.4 - 0.6	Negative	P < 0.05
EV	MDA	0.249723668	0.288313881	< 0.4	Positive	P >= 0.05
DNAI	MDA	-0.416134129	0.068004493	0.4 - 0.6	Negative	P >= 0.05
MI	MDA	-0.229663409	0.330028674	< 0.4	Negative	P >= 0.05
PMI	MDA	-0.446833723	0.048250657	0.4 - 0.6	Negative	P < 0.05
SAb	MDA	0.486803659	0.029500602	0.4 - 0.6	Positive	P < 0.05
SM	FSH	0.410680736	0.072069537	0.4 - 0.6	Positive	P >= 0.05
SD	FSH	0.061040009	0.798228672	< 0.4	Positive	P >= 0.05
EV	FSH	-0.34311711	0.138591128	< 0.4	Negative	P >= 0.05
DNAI	FSH	0.56111324	0.01005212	0.4 - 0.6	Positive	P < 0.05
MI	FSH	0.555307093	0.011029909	0.4 - 0.6	Positive	P < 0.05
PMI	FSH	0.189616253	0.423310075	< 0.4	Positive	P >= 0.05
SAb	FSH	-0.118133935	0.619868769	< 0.4	Negative	P >= 0.05
SM	LH	0.628055704	0.003024815	>= 0.6	Positive	P < 0.05
SD	LH	0.18952546	0.423536248	< 0.4	Positive	P >= 0.05
EV	LH	-0.274645846	0.241248197	< 0.4	Negative	P >= 0.05
DNAI	LH	0.828130935	6.53941E-06	>= 0.6	Positive	P < 0.05
MI	LH	0.364290513	0.114317832	< 0.4	Positive	P >= 0.05
PMI	LH	0.178329571	0.451916599	< 0.4	Positive	P >= 0.05

SAb	LH	-0.494356659	0.026713253	0.4 - 0.6	Negative	P < 0.05
SM	T	0.790522809	3.35087E-05	>= 0.6	Positive	P < 0.05
SD	T	0.385079563	0.093617108	< 0.4	Positive	P >= 0.05
EV	T	-0.390286203	0.088893741	< 0.4	Negative	P >= 0.05
DNAI	T	0.814592009	1.2276E-05	>= 0.6	Positive	P < 0.05
MI	T	0.606270015	0.004600786	>= 0.6	Positive	P < 0.05
PMI	T	0.283671934	0.22550242	< 0.4	Positive	P >= 0.05
SAb	T	-0.693754703	0.000692274	>= 0.6	Negative	P < 0.05
SM	T3	0.45112782	0.045883259	0.4 - 0.6	Positive	P < 0.05
SD	T3	0.385688066	0.093055798	< 0.4	Positive	P >= 0.05
EV	T3	-0.178718852	0.450913656	< 0.4	Negative	P >= 0.05
DNAI	T3	0.517293233	0.019500547	0.4 - 0.6	Positive	P < 0.05
MI	T3	0.51924898	0.018965881	0.4 - 0.6	Positive	P < 0.05
PMI	T3	0.030838663	0.89730661	< 0.4	Positive	P >= 0.05
SAb	T3	-0.533283227	0.015463218	0.4 - 0.6	Negative	P < 0.05
SM	T4	0.084242202	0.724003749	< 0.4	Positive	P >= 0.05
SD	T4	-0.148455331	0.532207265	< 0.4	Negative	P >= 0.05
EV	T4	0.292524454	0.210725851	< 0.4	Positive	P >= 0.05
DNAI	T4	0.26250472	0.263517038	< 0.4	Positive	P >= 0.05
MI	T4	0.274822273	0.240933822	< 0.4	Positive	P >= 0.05
PMI	T4	0.341610233	0.140446682	< 0.4	Positive	P >= 0.05
SAb	T4	-0.14221219	0.549777255	< 0.4	Negative	P >= 0.05
SM	COR	-0.548326477	0.012306094	0.4 - 0.6	Negative	P < 0.05
SD	COR	-0.085154581	0.721128284	< 0.4	Negative	P >= 0.05
EV	COR	0.36822324	0.110170085	< 0.4	Positive	P >= 0.05
DNAI	COR	-0.566378378	0.009227472	0.4 - 0.6	Negative	P < 0.05
MI	COR	-0.371463073	0.106835587	< 0.4	Negative	P >= 0.05
PMI	COR	-0.474793078	0.034397733	0.4 - 0.6	Negative	P < 0.05
SAb	COR	0.379984951	0.098414303	< 0.4	Positive	P >= 0.05

Table S10. B: The r values, p values, and rd, r.sign and pd of Figure 10.B

sepc	env	r	p	rd	r.sign	pd
SM	TSOD	0.048890564	0.837815471	< 0.4	Positive	P >= 0.05
SD	TSOD	-0.181612866	0.443493734	< 0.4	Negative	P >= 0.05
EV	TSOD	0.067710472	0.77668781	< 0.4	Positive	P >= 0.05
DNAI	TSOD	-0.064685977	0.78643634	< 0.4	Negative	P >= 0.05
MI	TSOD	0.045300375	0.849590083	< 0.4	Positive	P >= 0.05
PMI	TSOD	-0.108352144	0.649319746	< 0.4	Negative	P >= 0.05
SAb	TSOD	0.328066215	0.157912476	< 0.4	Positive	P >= 0.05
SM	TAOC	0.386207207	0.092578872	< 0.4	Positive	P >= 0.05
SD	TAOC	0.177503678	0.454048242	< 0.4	Positive	P >= 0.05
EV	TAOC	0.013814311	0.95390479	< 0.4	Positive	P >= 0.05
DNAI	TAOC	0.583483972	0.006919663	0.4 - 0.6	Positive	P < 0.05
MI	TAOC	0.082636361	0.729073376	< 0.4	Positive	P >= 0.05
PMI	TAOC	0.236441645	0.315552992	< 0.4	Positive	P >= 0.05
SAb	TAOC	-0.159019341	0.503083233	< 0.4	Negative	P >= 0.05
SM	CAT	-0.106887535	0.653774194	< 0.4	Negative	P >= 0.05
SD	CAT	-0.039969845	0.867128427	< 0.4	Negative	P >= 0.05
EV	CAT	-0.16407414	0.489425737	< 0.4	Negative	P >= 0.05
DNAI	CAT	-0.398946434	0.08142923	< 0.4	Negative	P >= 0.05
MI	CAT	-0.473745518	0.034853191	0.4 - 0.6	Negative	P < 0.05
PMI	CAT	-0.118975937	0.617358727	< 0.4	Negative	P >= 0.05
SAb	CAT	0.198795237	0.400780277	< 0.4	Positive	P >= 0.05
SM	MDA	-0.385225615	0.093482157	< 0.4	Negative	P >= 0.05
SD	MDA	-0.299093741	0.200183877	< 0.4	Negative	P >= 0.05
EV	MDA	0.361050861	0.117817996	< 0.4	Positive	P >= 0.05
DNAI	MDA	-0.26611476	0.2567648	< 0.4	Negative	P >= 0.05
MI	MDA	-0.349224591	0.131247063	< 0.4	Negative	P >= 0.05
PMI	MDA	-0.136878178	0.564992269	< 0.4	Negative	P >= 0.05
SAb	MDA	0.521494547	0.018366479	0.4 - 0.6	Positive	P < 0.05
SM	FSH	0.434749937	0.055416597	0.4 - 0.6	Positive	P >= 0.05
SD	FSH	-0.029012844	0.903358508	< 0.4	Negative	P >= 0.05
EV	FSH	-0.657324131	0.001636527	>= 0.6	Negative	P < 0.05
DNAI	FSH	0.335464484	0.148195206	< 0.4	Positive	P >= 0.05
MI	FSH	0.460931313	0.040813512	0.4 - 0.6	Positive	P < 0.05
PMI	FSH	0.203160271	0.390302154	< 0.4	Positive	P >= 0.05
SAb	FSH	-0.341610233	0.140446682	< 0.4	Negative	P >= 0.05
SM	LH	0.70052691	0.000582054	>= 0.6	Positive	P < 0.05
SD	LH	0.256690703	0.274624653	< 0.4	Positive	P >= 0.05
EV	LH	-0.378255869	0.100082476	< 0.4	Negative	P >= 0.05
DNAI	LH	0.605718757	0.004648087	>= 0.6	Positive	P < 0.05
MI	LH	0.465636566	0.03853978	0.4 - 0.6	Positive	P < 0.05
PMI	LH	0.203989476	0.388329032	< 0.4	Positive	P >= 0.05
SAb	LH	-0.558901056	0.010415999	0.4 - 0.6	Negative	P < 0.05

SM	T	0.579917303	0.00735692	0.4 - 0.6	Positive	P < 0.05
SD	T	0.23097238	0.327203097	< 0.4	Positive	P >= 0.05
EV	T	-0.454192715	0.044248926	0.4 - 0.6	Negative	P < 0.05
DNAI	T	0.617525429	0.003718497	>= 0.6	Positive	P < 0.05
MI	T	0.401663322	0.079186131	0.4 - 0.6	Positive	P >= 0.05
PMI	T	0.082016554	0.731033014	< 0.4	Positive	P >= 0.05
SAb	T	-0.453724605	0.044495596	0.4 - 0.6	Negative	P < 0.05
SM	T3	0.455810487	0.043404548	0.4 - 0.6	Positive	P < 0.05
SD	T3	0.097965447	0.681149032	< 0.4	Positive	P >= 0.05
EV	T3	-0.132377776	0.577971386	< 0.4	Negative	P >= 0.05
DNAI	T3	0.616773267	0.003772709	>= 0.6	Positive	P < 0.05
MI	T3	0.472633909	0.035341623	0.4 - 0.6	Positive	P < 0.05
PMI	T3	0.262603461	0.263330876	< 0.4	Positive	P >= 0.05
SAb	T3	-0.374717833	0.103559893	< 0.4	Negative	P >= 0.05
SM	T4	0.267017695	0.255093269	< 0.4	Positive	P >= 0.05
SD	T4	-0.135644465	0.568537515	< 0.4	Negative	P >= 0.05
EV	T4	-0.140746486	0.553939653	< 0.4	Negative	P >= 0.05
DNAI	T4	0.567882703	0.00900226	0.4 - 0.6	Positive	P < 0.05
MI	T4	0.358627966	0.120485448	< 0.4	Positive	P >= 0.05
PMI	T4	0.118510158	0.618746731	< 0.4	Positive	P >= 0.05
SAb	T4	-0.189616253	0.423310075	< 0.4	Negative	P >= 0.05
SM	COR	-0.584430278	0.006807297	0.4 - 0.6	Negative	P < 0.05
SD	COR	-0.123963969	0.6025736	< 0.4	Negative	P >= 0.05
EV	COR	-0.005325543	0.982222177	< 0.4	Negative	P >= 0.05
DNAI	COR	-0.549830802	0.012021459	0.4 - 0.6	Negative	P < 0.05
MI	COR	-0.484336506	0.030458912	0.4 - 0.6	Negative	P < 0.05
PMI	COR	-0.554176072	0.011229061	0.4 - 0.6	Negative	P < 0.05
SAb	COR	0.47103085	0.036055365	0.4 - 0.6	Positive	P < 0.05
