# Supplementary material for: Melatonin Improves Semen Quality by Modulating Oxidative Stress, Endocrine Hormones, and Tryptophan Metabolism of Hu Rams Under Summer Heat Stress and the Non-Reproductive Season
Source: Antioxidants (Basel). 2025 May 24;14(6):630. doi: 10.3390/antiox14060630 (PMC12189995; doi:10.3390/antiox14060630)
Supplement: Supplementary file 1 [file antioxidants-14-00630-s001.zip › Table S11The r values, p values, and rd, r.sign and pd of Figure 11.pdf]

Table S11. A: The r values, p values, and rd, r.sign and pd of Figure 11.A

sepc	env	r	p	rd	r.sign	pd
SM	TRP	-0.032967033	0.910918653	< 0.4	Negative	P >= 0.05
SD	TRP	-0.301430325	0.294956934	< 0.4	Negative	P >= 0.05
EV	TRP	0.193562877	0.507306588	< 0.4	Positive	P >= 0.05
DNAI	TRP	0.375824176	0.185410029	< 0.4	Positive	P >= 0.05
MI	TRP	0.381902177	0.177827764	< 0.4	Positive	P >= 0.05
PMI	TRP	0.024175824	0.934618995	< 0.4	Positive	P >= 0.05
SAb	TRP	0.221978022	0.445629658	< 0.4	Positive	P >= 0.05
SM	5-HT	-0.692307692	0.006071049	>= 0.6	Negative	P < 0.05
SD	5-HT	-0.396039844	0.160975271	< 0.4	Negative	P >= 0.05
EV	5-HT	-0.03337291	0.909825963	< 0.4	Negative	P >= 0.05
DNAI	5-HT	-0.679120879	0.007562257	>= 0.6	Negative	P < 0.05
MI	5-HT	-0.403977447	0.151989231	0.4 - 0.6	Negative	P >= 0.05
PMI	5-HT	-0.296703297	0.302966724	< 0.4	Negative	P >= 0.05
SAb	5-HT	0.758241758	0.001673003	>= 0.6	Positive	P < 0.05
SM	5-HTP	-0.67032967	0.008704234	>= 0.6	Negative	P < 0.05
SD	5-HTP	-0.431243385	0.123670191	0.4 - 0.6	Negative	P >= 0.05
EV	5-HTP	0.177988853	0.54267329	< 0.4	Positive	P >= 0.05
DNAI	5-HTP	-0.441758242	0.113777503	0.4 - 0.6	Negative	P >= 0.05
MI	5-HTP	-0.346581744	0.224762433	< 0.4	Negative	P >= 0.05
PMI	5-HTP	-0.191208791	0.51258397	< 0.4	Negative	P >= 0.05
SAb	5-HTP	0.661538462	0.009975179	>= 0.6	Positive	P < 0.05
SM	NAS	-0.810989011	0.00043221	>= 0.6	Negative	P < 0.05
SD	NAS	-0.561056445	0.036851588	0.4 - 0.6	Negative	P < 0.05
EV	NAS	0.048946934	0.868026677	< 0.4	Positive	P >= 0.05
DNAI	NAS	-0.520879121	0.056153927	0.4 - 0.6	Negative	P >= 0.05
MI	NAS	-0.289186041	0.315959935	< 0.4	Negative	P >= 0.05
PMI	NAS	-0.314285714	0.273804903	< 0.4	Negative	P >= 0.05
SAb	NAS	0.863736264	6.84638E-05	>= 0.6	Positive	P < 0.05
SM	MT	0.745054945	0.002230037	>= 0.6	Positive	P < 0.05
SD	MT	0.613861758	0.019534046	>= 0.6	Positive	P < 0.05
EV	MT	-0.378226312	0.182389013	< 0.4	Negative	P >= 0.05
DNAI	MT	0.415384615	0.139665948	0.4 - 0.6	Positive	P >= 0.05
MI	MT	0.169979582	0.561265469	< 0.4	Positive	P >= 0.05
PMI	MT	-0.072527473	0.805376314	< 0.4	Negative	P >= 0.05
SAb	MT	-0.578021978	0.030383014	0.4 - 0.6	Negative	P < 0.05
SM	3-HAA	-0.41978022	0.135101074	0.4 - 0.6	Negative	P >= 0.05
SD	3-HAA	-0.396039844	0.160975271	< 0.4	Negative	P >= 0.05
EV	3-HAA	-0.10234359	0.727728786	< 0.4	Negative	P >= 0.05
DNAI	3-HAA	-0.147252747	0.615418762	< 0.4	Negative	P >= 0.05
MI	3-HAA	-0.002207527	0.994024204	< 0.4	Negative	P >= 0.05
PMI	3-HAA	-0.331868132	0.246377183	< 0.4	Negative	P >= 0.05

SAb	3-HAA	0.406593407	0.149101939	0.4 - 0.6	Positive	P >= 0.05
SM	3-HK	-0.569230769	0.033619417	0.4 - 0.6	Negative	P < 0.05
SD	3-HK	-0.605060872	0.021871945	>= 0.6	Negative	P < 0.05
EV	3-HK	0.160189967	0.584345934	< 0.4	Positive	P >= 0.05
DNAI	3-HK	-0.243956044	0.400624804	< 0.4	Negative	P >= 0.05
MI	3-HK	0.099338717	0.735457787	< 0.4	Positive	P >= 0.05
PMI	3-HK	-0.125274725	0.669582397	< 0.4	Negative	P >= 0.05
SAb	3-HK	0.472527473	0.087967425	0.4 - 0.6	Positive	P >= 0.05
SM	5-HIAA	-0.569230769	0.033619417	0.4 - 0.6	Negative	P < 0.05
SD	5-HIAA	-0.24422457	0.40009013	< 0.4	Negative	P >= 0.05
EV	5-HIAA	0.204687181	0.482708779	< 0.4	Positive	P >= 0.05
DNAI	5-HIAA	-0.323076923	0.25987362	< 0.4	Negative	P >= 0.05
MI	5-HIAA	-0.350996798	0.218514189	< 0.4	Negative	P >= 0.05
PMI	5-HIAA	-0.230769231	0.427335862	< 0.4	Negative	P >= 0.05
SAb	5-HIAA	0.578021978	0.030383014	0.4 - 0.6	Positive	P < 0.05
SM	5-HTOL	-0.204395604	0.483346223	< 0.4	Negative	P >= 0.05
SD	5-HTOL	-0.415841836	0.139186387	0.4 - 0.6	Negative	P >= 0.05
EV	5-HTOL	-0.111243033	0.704981197	< 0.4	Negative	P >= 0.05
DNAI	5-HTOL	0.358241758	0.208498125	< 0.4	Positive	P >= 0.05
MI	5-HTOL	0.147904311	0.613838846	< 0.4	Positive	P >= 0.05
PMI	5-HTOL	0.305494505	0.288169886	< 0.4	Positive	P >= 0.05
SAb	5-HTOL	0.261538462	0.366411137	< 0.4	Positive	P >= 0.05
SM	5-Me-IAA	-0.037362637	0.899093097	< 0.4	Negative	P >= 0.05
SD	5-Me-IAA	-0.550055338	0.041556212	0.4 - 0.6	Negative	P < 0.05
EV	5-Me-IAA	0.082319844	0.779654081	< 0.4	Positive	P >= 0.05
DNAI	5-Me-IAA	0.195604396	0.502750091	< 0.4	Positive	P >= 0.05
MI	5-Me-IAA	0.452543042	0.104207008	0.4 - 0.6	Positive	P >= 0.05
PMI	5-Me-IAA	0.142857143	0.626117476	< 0.4	Positive	P >= 0.05
SAb	5-Me-IAA	0.389010989	0.16921739	< 0.4	Positive	P >= 0.05
SM	AA	0.705494505	0.004819558	>= 0.6	Positive	P < 0.05
SD	AA	0.224422578	0.440504282	< 0.4	Positive	P >= 0.05
EV	AA	-0.224710927	0.439901659	< 0.4	Negative	P >= 0.05
DNAI	AA	0.362637363	0.202564544	< 0.4	Positive	P >= 0.05
MI	AA	-0.006622581	0.982073776	< 0.4	Negative	P >= 0.05
PMI	AA	0.340659341	0.233316051	< 0.4	Positive	P >= 0.05
SAb	AA	-0.49010989	0.075220067	0.4 - 0.6	Negative	P >= 0.05
SM	IA	0.327472527	0.253071001	< 0.4	Positive	P >= 0.05
SD	IA	-0.193619479	0.507180004	< 0.4	Negative	P >= 0.05
EV	IA	0.109018172	0.710647463	< 0.4	Positive	P >= 0.05
DNAI	IA	0.292307692	0.31052632	< 0.4	Positive	P >= 0.05
MI	IA	0.359826907	0.206345892	< 0.4	Positive	P >= 0.05
PMI	IA	0.696703297	0.005628677	>= 0.6	Positive	P < 0.05
SAb	IA	-0.27032967	0.349919386	< 0.4	Negative	P >= 0.05
SM	IAA	-0.142857143	0.626117476	< 0.4	Negative	P >= 0.05

SD	IAA	-0.723872825	0.003422322	>= 0.6	Negative	P < 0.05
EV	IAA	-0.006674582	0.98193304	< 0.4	Negative	P >= 0.05
DNAI	IAA	0.393406593	0.164031573	< 0.4	Positive	P >= 0.05
MI	IAA	0.29801615	0.30072967	< 0.4	Positive	P >= 0.05
PMI	IAA	0.450549451	0.105932785	0.4 - 0.6	Positive	P >= 0.05
SAb	IAA	0.367032967	0.196738872	< 0.4	Positive	P >= 0.05
SM	IAA-Ala	-0.608791209	0.020856616	>= 0.6	Negative	P < 0.05
SD	IAA-Ala	-0.29262944	0.309969343	< 0.4	Negative	P >= 0.05
EV	IAA-Ala	0.611836681	0.020054412	>= 0.6	Positive	P < 0.05
DNAI	IAA-Ala	-0.265934066	0.3581134	< 0.4	Negative	P >= 0.05
MI	IAA-Ala	-0.231790339	0.425236003	< 0.4	Negative	P >= 0.05
PMI	IAA-Ala	-0.046153846	0.875502538	< 0.4	Negative	P >= 0.05
SAb	IAA-Ala	0.569230769	0.033619417	0.4 - 0.6	Positive	P < 0.05
SM	IAA-Asp	0.023052308	0.937651917	< 0.4	Positive	P >= 0.05
SD	IAA-Asp	-0.110260035	0.707482959	< 0.4	Negative	P >= 0.05
EV	IAA-Asp	0.103716082	0.724206465	< 0.4	Positive	P >= 0.05
DNAI	IAA-Asp	-0.099893333	0.734029452	< 0.4	Negative	P >= 0.05
MI	IAA-Asp	-0.239261199	0.410032926	< 0.4	Negative	P >= 0.05
PMI	IAA-Asp	-0.053788718	0.855091954	< 0.4	Negative	P >= 0.05
SAb	IAA-Asp	-0.058911453	0.84144347	< 0.4	Negative	P >= 0.05
SM	IAM	-0.076923077	0.793805888	< 0.4	Negative	P >= 0.05
SD	IAM	-0.228823021	0.431352629	< 0.4	Negative	P >= 0.05
EV	IAM	-0.467220738	0.092094332	0.4 - 0.6	Negative	P >= 0.05
DNAI	IAM	-0.046153846	0.875502538	< 0.4	Negative	P >= 0.05
MI	IAM	0.390732285	0.167174106	< 0.4	Positive	P >= 0.05
PMI	IAM	0.16043956	0.583752753	< 0.4	Positive	P >= 0.05
SAb	IAM	-0.081318681	0.782274751	< 0.4	Negative	P >= 0.05
SM	IAN	-0.030510632	0.917534765	< 0.4	Negative	P >= 0.05
SD	IAN	0.143322858	0.624980655	< 0.4	Positive	P >= 0.05
EV	IAN	-0.458542269	0.099130774	0.4 - 0.6	Negative	P >= 0.05
DNAI	IAN	0.007040915	0.980941597	< 0.4	Positive	P >= 0.05
MI	IAN	-0.247522442	0.39355406	< 0.4	Negative	P >= 0.05
PMI	IAN	-0.298065407	0.300645924	< 0.4	Negative	P >= 0.05
SAb	IAN	-0.044592462	0.879685919	< 0.4	Negative	P >= 0.05
SM	ICA	-0.010989011	0.97025839	< 0.4	Negative	P >= 0.05
SD	ICA	-0.382838515	0.176677787	< 0.4	Negative	P >= 0.05
EV	ICA	-0.002224861	0.993977282	< 0.4	Negative	P >= 0.05
DNAI	ICA	0.397802198	0.158950775	< 0.4	Positive	P >= 0.05
MI	ICA	0.637975313	0.014091563	>= 0.6	Positive	P < 0.05
PMI	ICA	0.107692308	0.714030809	< 0.4	Positive	P >= 0.05
SAb	ICA	0.151648352	0.604790598	< 0.4	Positive	P >= 0.05
SM	IE	0.27459569	0.342066733	< 0.4	Positive	P >= 0.05
SD	IE	-0.166818409	0.568676369	< 0.4	Negative	P >= 0.05
EV	IE	-0.532194137	0.050111898	0.4 - 0.6	Negative	P >= 0.05

DNAI	IE	0.448271596	0.107928586	0.4 - 0.6	Positive	P >= 0.05
MI	IE	0.667131914	0.009151117	>= 0.6	Positive	P < 0.05
PMI	IE	0.166634991	0.569107608	< 0.4	Positive	P >= 0.05
SAb	IE	-0.161941048	0.580189482	< 0.4	Negative	P >= 0.05
SM	IGA	-0.607261094	0.021268686	>= 0.6	Negative	P < 0.05
SD	IGA	-0.45814978	0.099457538	0.4 - 0.6	Negative	P >= 0.05
EV	IGA	0.086865074	0.767784076	< 0.4	Positive	P >= 0.05
DNAI	IGA	-0.206820807	0.478056349	< 0.4	Negative	P >= 0.05
MI	IGA	0.013259741	0.964115982	< 0.4	Positive	P >= 0.05
PMI	IGA	-0.125412617	0.669237535	< 0.4	Negative	P >= 0.05
SAb	IGA	0.732673711	0.00287784	>= 0.6	Positive	P < 0.05
SM	ILA	0.182417582	0.532508819	< 0.4	Positive	P >= 0.05
SD	ILA	-0.288228997	0.317636568	< 0.4	Negative	P >= 0.05
EV	ILA	0.313705353	0.274739869	< 0.4	Positive	P >= 0.05
DNAI	ILA	0.397802198	0.158950775	< 0.4	Positive	P >= 0.05
MI	ILA	0.554089285	0.039782703	0.4 - 0.6	Positive	P < 0.05
PMI	ILA	0.358241758	0.208498125	< 0.4	Positive	P >= 0.05
SAb	ILA	0.002197802	0.994050529	< 0.4	Positive	P >= 0.05
SM	Indican	0.125274725	0.669582397	< 0.4	Positive	P >= 0.05
SD	Indican	-0.059405977	0.840128042	< 0.4	Negative	P >= 0.05
EV	Indican	0.418273804	0.136654074	0.4 - 0.6	Positive	P >= 0.05
DNAI	Indican	0.151648352	0.604790598	< 0.4	Positive	P >= 0.05
MI	Indican	-0.103753771	0.724109813	< 0.4	Negative	P >= 0.05
PMI	Indican	0.195604396	0.502750091	< 0.4	Positive	P >= 0.05
SAb	Indican	0.081318681	0.782274751	< 0.4	Positive	P >= 0.05
SM	IPA	-0.27032967	0.349919386	< 0.4	Negative	P >= 0.05
SD	IPA	-0.308030989	0.283980809	< 0.4	Negative	P >= 0.05
EV	IPA	0.558440025	0.037932981	0.4 - 0.6	Positive	P < 0.05
DNAI	IPA	-0.217582418	0.454919373	< 0.4	Negative	P >= 0.05
MI	IPA	0.041943014	0.886791155	< 0.4	Positive	P >= 0.05
PMI	IPA	0.195604396	0.502750091	< 0.4	Positive	P >= 0.05
SAb	IPA	0.283516484	0.325965947	< 0.4	Positive	P >= 0.05
SM	IS	-0.652747253	0.011384574	>= 0.6	Negative	P < 0.05
SD	IS	-0.495049805	0.071887735	0.4 - 0.6	Negative	P >= 0.05
EV	IS	0.200237459	0.492479735	< 0.4	Positive	P >= 0.05
DNAI	IS	-0.173626374	0.552766941	< 0.4	Negative	P >= 0.05
MI	IS	0.346581744	0.224762433	< 0.4	Positive	P >= 0.05
PMI	IS	-0.085714286	0.770785031	< 0.4	Negative	P >= 0.05
SAb	IS	0.727472527	0.003190616	>= 0.6	Positive	P < 0.05
SM	KYN	-0.327472527	0.253071001	< 0.4	Negative	P >= 0.05
SD	KYN	-0.514851797	0.059578787	0.4 - 0.6	Negative	P >= 0.05
EV	KYN	-0.062296098	0.832448198	< 0.4	Negative	P >= 0.05
DNAI	KYN	-0.279120879	0.333845077	< 0.4	Negative	P >= 0.05
MI	KYN	0.147904311	0.613838846	< 0.4	Positive	P >= 0.05

PMI	KYN	0.006593407	0.982152735	< 0.4	Positive	P >= 0.05
SAb	KYN	0.432967033	0.122010006	0.4 - 0.6	Positive	P >= 0.05
SM	KYNA	0.23956044	0.409429893	< 0.4	Positive	P >= 0.05
SD	KYNA	0.105610625	0.719352701	< 0.4	Positive	P >= 0.05
EV	KYNA	0.051171795	0.862079008	< 0.4	Positive	P >= 0.05
DNAI	KYNA	0.006593407	0.982152735	< 0.4	Positive	P >= 0.05
MI	KYNA	-0.116998933	0.690388522	< 0.4	Negative	P >= 0.05
PMI	KYNA	-0.437362637	0.117844832	0.4 - 0.6	Negative	P >= 0.05
SAb	KYNA	-0.010989011	0.97025839	< 0.4	Negative	P >= 0.05
SM	NiA	-0.296703297	0.302966724	< 0.4	Negative	P >= 0.05
SD	NiA	0.077007747	0.793583392	< 0.4	Positive	P >= 0.05
EV	NiA	-0.569564329	0.033492151	0.4 - 0.6	Negative	P < 0.05
DNAI	NiA	0.028571429	0.922761377	< 0.4	Positive	P >= 0.05
MI	NiA	0.183224744	0.530665355	< 0.4	Positive	P >= 0.05
PMI	NiA	-0.345054945	0.22694868	< 0.4	Negative	P >= 0.05
SAb	NiA	-0.032967033	0.910918653	< 0.4	Negative	P >= 0.05
SM	skatole	0.164835165	0.573346405	< 0.4	Positive	P >= 0.05
SD	skatole	0.127612838	0.663743109	< 0.4	Positive	P >= 0.05
EV	skatole	-0.137941361	0.638163693	< 0.4	Negative	P >= 0.05
DNAI	skatole	0.191208791	0.51258397	< 0.4	Positive	P >= 0.05
MI	skatole	-0.011037635	0.970126843	< 0.4	Negative	P >= 0.05
PMI	skatole	-0.257142857	0.374811904	< 0.4	Negative	P >= 0.05
SAb	skatole	0.182417582	0.532508819	< 0.4	Positive	P >= 0.05
SM	Xa	-0.147252747	0.615418762	< 0.4	Negative	P >= 0.05
SD	Xa	-0.426842943	0.127977799	0.4 - 0.6	Negative	P >= 0.05
EV	Xa	0.097893869	0.739182507	< 0.4	Positive	P >= 0.05
DNAI	Xa	0.279120879	0.333845077	< 0.4	Positive	P >= 0.05
MI	Xa	0.275940879	0.339611058	< 0.4	Positive	P >= 0.05
PMI	Xa	0.120879121	0.68060702	< 0.4	Positive	P >= 0.05
SAb	Xa	0.27032967	0.349919386	< 0.4	Positive	P >= 0.05

Table S11. B: The r values, p values, and rd, r.sign and pd of Figure 11.B

sepc	env	r	p	rd	r.sign	pd
SM	TRP	0.701098901	0.005211862	>= 0.6	Positive	P < 0.05
SD	TRP	0.512651575	0.06086563	0.4 - 0.6	Positive	P >= 0.05
EV	TRP	0.213586623	0.463445487	< 0.4	Positive	P >= 0.05
DNAI	TRP	0.23956044	0.409429893	< 0.4	Positive	P >= 0.05
MI	TRP	0.22296023	0.443566775	< 0.4	Positive	P >= 0.05
PMI	TRP	0.147252747	0.615418762	< 0.4	Positive	P >= 0.05
SAb	TRP	-0.432967033	0.122010006	0.4 - 0.6	Negative	P >= 0.05
SM	5-HT	0.032967033	0.910918653	< 0.4	Positive	P >= 0.05
SD	5-HT	-0.459846263	0.098050482	0.4 - 0.6	Negative	P >= 0.05
EV	5-HT	0.200237459	0.492479735	< 0.4	Positive	P >= 0.05
DNAI	5-HT	0.221978022	0.445629658	< 0.4	Positive	P >= 0.05
MI	5-HT	0.434882826	0.120182559	0.4 - 0.6	Positive	P >= 0.05
PMI	5-HT	0.367032967	0.196738872	< 0.4	Positive	P >= 0.05
SAb	5-HT	0.415384615	0.139665948	0.4 - 0.6	Positive	P >= 0.05
SM	5-HTP	0.806593407	0.000491113	>= 0.6	Positive	P < 0.05
SD	5-HTP	0.200220143	0.492517938	< 0.4	Positive	P >= 0.05
EV	5-HTP	-0.008899443	0.97591211	< 0.4	Negative	P >= 0.05
DNAI	5-HTP	0.56043956	0.037104477	0.4 - 0.6	Positive	P < 0.05
MI	5-HTP	0.417222609	0.137744827	0.4 - 0.6	Positive	P >= 0.05
PMI	5-HTP	0.358241758	0.208498125	< 0.4	Positive	P >= 0.05
SAb	5-HTP	-0.578021978	0.030383014	0.4 - 0.6	Negative	P < 0.05
SM	NAS	0.525274725	0.053747502	0.4 - 0.6	Positive	P >= 0.05
SD	NAS	-0.057205755	0.845983544	< 0.4	Negative	P >= 0.05
EV	NAS	-0.47389532	0.086925014	0.4 - 0.6	Negative	P >= 0.05
DNAI	NAS	0.393406593	0.164031573	< 0.4	Positive	P >= 0.05
MI	NAS	0.275940879	0.339611058	< 0.4	Positive	P >= 0.05
PMI	NAS	0.318681319	0.266784961	< 0.4	Positive	P >= 0.05
SAb	NAS	-0.534065934	0.049159916	0.4 - 0.6	Negative	P < 0.05
SM	MT	0.696703297	0.005628677	>= 0.6	Positive	P < 0.05
SD	MT	0.477448034	0.084258016	0.4 - 0.6	Positive	P >= 0.05
EV	MT	-0.213586623	0.463445487	< 0.4	Negative	P >= 0.05
DNAI	MT	0.582417582	0.028854655	0.4 - 0.6	Positive	P < 0.05
MI	MT	0.280355933	0.331620556	< 0.4	Positive	P >= 0.05
PMI	MT	0.134065934	0.647719528	< 0.4	Positive	P >= 0.05
SAb	MT	-0.789010989	0.000794614	>= 0.6	Negative	P < 0.05
SM	3-HAA	0.41978022	0.135101074	0.4 - 0.6	Positive	P >= 0.05
SD	3-HAA	-0.25962612	0.370053337	< 0.4	Negative	P >= 0.05
EV	3-HAA	0.173539131	0.552969609	< 0.4	Positive	P >= 0.05
DNAI	3-HAA	0.665934066	0.009322999	>= 0.6	Positive	P < 0.05
MI	3-HAA	0.540844123	0.045822463	0.4 - 0.6	Positive	P < 0.05
PMI	3-HAA	0.459340659	0.098468376	0.4 - 0.6	Positive	P >= 0.05

SAb	3-HAA	-0.371428571	0.191020812	< 0.4	Negative	P >= 0.05
SM	3-HK	0.12967033	0.658619276	< 0.4	Positive	P >= 0.05
SD	3-HK	-0.440044271	0.115351888	0.4 - 0.6	Negative	P >= 0.05
EV	3-HK	0.122367336	0.676867655	< 0.4	Positive	P >= 0.05
DNAI	3-HK	0.318681319	0.266784961	< 0.4	Positive	P >= 0.05
MI	3-HK	0.651220475	0.01164417	>= 0.6	Positive	P < 0.05
PMI	3-HK	0.16043956	0.583752753	< 0.4	Positive	P >= 0.05
SAb	3-HK	0.041758242	0.887286973	< 0.4	Positive	P >= 0.05
SM	5-HIAA	-0.024175824	0.934618995	< 0.4	Negative	P >= 0.05
SD	5-HIAA	0.077007747	0.793583392	< 0.4	Positive	P >= 0.05
EV	5-HIAA	0.035597771	0.903838935	< 0.4	Positive	P >= 0.05
DNAI	5-HIAA	0.257142857	0.374811904	< 0.4	Positive	P >= 0.05
MI	5-HIAA	0.249450555	0.389758912	< 0.4	Positive	P >= 0.05
PMI	5-HIAA	-0.226373626	0.436434795	< 0.4	Negative	P >= 0.05
SAb	5-HIAA	-0.169230769	0.563017274	< 0.4	Negative	P >= 0.05
SM	5-Me-IAA	-0.032967033	0.910918653	< 0.4	Negative	P >= 0.05
SD	5-Me-IAA	-0.286028776	0.321510256	< 0.4	Negative	P >= 0.05
EV	5-Me-IAA	0.091219287	0.756457086	< 0.4	Positive	P >= 0.05
DNAI	5-Me-IAA	0.01978022	0.946489227	< 0.4	Positive	P >= 0.05
MI	5-Me-IAA	0.05960323	0.839603457	< 0.4	Positive	P >= 0.05
PMI	5-Me-IAA	-0.164835165	0.573346405	< 0.4	Negative	P >= 0.05
SAb	5-Me-IAA	0.12967033	0.658619276	< 0.4	Positive	P >= 0.05
SM	AA	0.52967033	0.051416598	0.4 - 0.6	Positive	P >= 0.05
SD	AA	0.085808633	0.770538886	< 0.4	Positive	P >= 0.05
EV	AA	0.048946934	0.868026677	< 0.4	Positive	P >= 0.05
DNAI	AA	0.336263736	0.239792202	< 0.4	Positive	P >= 0.05
MI	AA	0.187639798	0.520631995	< 0.4	Positive	P >= 0.05
PMI	AA	0.30989011	0.280933281	< 0.4	Positive	P >= 0.05
SAb	AA	-0.314285714	0.273804903	< 0.4	Negative	P >= 0.05
SM	IA	0.6	0.023308411	0.4 - 0.6	Positive	P < 0.05
SD	IA	0.279428112	0.333290925	< 0.4	Positive	P >= 0.05
EV	IA	0.055621516	0.850204314	< 0.4	Positive	P >= 0.05
DNAI	IA	0.072527473	0.805376314	< 0.4	Positive	P >= 0.05
MI	IA	-0.108168825	0.712814265	< 0.4	Negative	P >= 0.05
PMI	IA	0.068131868	0.81698388	< 0.4	Positive	P >= 0.05
SAb	IA	-0.402197802	0.15397443	0.4 - 0.6	Negative	P >= 0.05
SM	IAA	-0.27032967	0.349919386	< 0.4	Negative	P >= 0.05
SD	IAA	0.09680974	0.74198082	< 0.4	Positive	P >= 0.05
EV	IAA	0.240284951	0.407971749	< 0.4	Positive	P >= 0.05
DNAI	IAA	-0.138461538	0.636884991	< 0.4	Negative	P >= 0.05
MI	IAA	-0.192054852	0.510684418	< 0.4	Negative	P >= 0.05
PMI	IAA	-0.494505495	0.072249674	0.4 - 0.6	Negative	P >= 0.05
SAb	IAA	0.147252747	0.615418762	< 0.4	Positive	P >= 0.05
SM	IAA-Ala	0.068131868	0.81698388	< 0.4	Positive	P >= 0.05

SD	IAA-Ala	-0.316831875	0.269725365	< 0.4	Negative	P >= 0.05
EV	IAA-Ala	0.13349164	0.649139938	< 0.4	Positive	P >= 0.05
DNAI	IAA-Ala	0.265934066	0.3581134	< 0.4	Positive	P >= 0.05
MI	IAA-Ala	0.271525825	0.347707628	< 0.4	Positive	P >= 0.05
PMI	IAA-Ala	-0.151648352	0.604790598	< 0.4	Negative	P >= 0.05
SAb	IAA-Ala	0.138461538	0.636884991	< 0.4	Positive	P >= 0.05
SM	IAA-Asp	0.107692308	0.714030809	< 0.4	Positive	P >= 0.05
SD	IAA-Asp	0.719472383	0.00372333	>= 0.6	Positive	P < 0.05
EV	IAA-Asp	0.262533558	0.364523612	< 0.4	Positive	P >= 0.05
DNAI	IAA-Asp	-0.186813187	0.522504012	< 0.4	Negative	P >= 0.05
MI	IAA-Asp	-0.55850434	0.037906122	0.4 - 0.6	Negative	P < 0.05
PMI	IAA-Asp	-0.235164835	0.418333896	< 0.4	Negative	P >= 0.05
SAb	IAA-Asp	-0.217582418	0.454919373	< 0.4	Negative	P >= 0.05
SM	IAM	-0.12967033	0.658619276	< 0.4	Negative	P >= 0.05
SD	IAM	0.29262944	0.309969343	< 0.4	Positive	P >= 0.05
EV	IAM	0.106793312	0.716327656	< 0.4	Positive	P >= 0.05
DNAI	IAM	-0.248351648	0.391919539	< 0.4	Negative	P >= 0.05
MI	IAM	-0.05960323	0.839603457	< 0.4	Negative	P >= 0.05
PMI	IAM	-0.446153846	0.109807128	0.4 - 0.6	Negative	P >= 0.05
SAb	IAM	-0.068131868	0.81698388	< 0.4	Negative	P >= 0.05
SM	IAN	0.296703297	0.302966724	< 0.4	Positive	P >= 0.05
SD	IAN	-0.437844049	0.117394618	0.4 - 0.6	Negative	P >= 0.05
EV	IAN	-0.193562877	0.507306588	< 0.4	Negative	P >= 0.05
DNAI	IAN	0.287912088	0.318192871	< 0.4	Positive	P >= 0.05
MI	IAN	0.258280663	0.372627516	< 0.4	Positive	P >= 0.05
PMI	IAN	0.059340659	0.840301763	< 0.4	Positive	P >= 0.05
SAb	IAN	0.006593407	0.982152735	< 0.4	Positive	P >= 0.05
SM	ICA	-0.248351648	0.391919539	< 0.4	Negative	P >= 0.05
SD	ICA	0.11441151	0.696936272	< 0.4	Positive	P >= 0.05
EV	ICA	0	1	< 0.4	Negative	P >= 0.05
DNAI	ICA	-0.191208791	0.51258397	< 0.4	Negative	P >= 0.05
MI	ICA	0.183224744	0.530665355	< 0.4	Positive	P >= 0.05
PMI	ICA	-0.591208791	0.025970814	0.4 - 0.6	Negative	P < 0.05
SAb	ICA	0.265934066	0.3581134	< 0.4	Positive	P >= 0.05
SM	IGA	0.002197802	0.994050529	< 0.4	Positive	P >= 0.05
SD	IGA	0.330033203	0.24915826	< 0.4	Positive	P >= 0.05
EV	IGA	0.213586623	0.463445487	< 0.4	Positive	P >= 0.05
DNAI	IGA	-0.2	0.493003735	< 0.4	Negative	P >= 0.05
MI	IGA	-0.514353799	0.059868322	0.4 - 0.6	Negative	P >= 0.05
PMI	IGA	0.024175824	0.934618995	< 0.4	Positive	P >= 0.05
SAb	IGA	-0.432967033	0.122010006	0.4 - 0.6	Negative	P >= 0.05
SM	ILA	0.30989011	0.280933281	< 0.4	Positive	P >= 0.05
SD	ILA	0.250825234	0.387064952	< 0.4	Positive	P >= 0.05
EV	ILA	0.071195541	0.80888975	< 0.4	Positive	P >= 0.05

DNAI	ILA	0.283516484	0.325965947	< 0.4	Positive	P >= 0.05
MI	ILA	-0.271525825	0.347707628	< 0.4	Negative	P >= 0.05
PMI	ILA	0.156043956	0.594234701	< 0.4	Positive	P >= 0.05
SAb	ILA	-0.30989011	0.280933281	< 0.4	Negative	P >= 0.05
SM	IPA	-0.024175824	0.934618995	< 0.4	Negative	P >= 0.05
SD	IPA	-0.121012174	0.680272424	< 0.4	Negative	P >= 0.05
EV	IPA	-0.031148049	0.915817442	< 0.4	Negative	P >= 0.05
DNAI	IPA	0.195604396	0.502750091	< 0.4	Positive	P >= 0.05
MI	IPA	0.315676366	0.271572246	< 0.4	Positive	P >= 0.05
PMI	IPA	0.024175824	0.934618995	< 0.4	Positive	P >= 0.05
SAb	IPA	-0.050549451	0.863742036	< 0.4	Negative	P >= 0.05
SM	IS	0.063736264	0.828626423	< 0.4	Positive	P >= 0.05
SD	IS	-0.187018815	0.522038048	< 0.4	Negative	P >= 0.05
EV	IS	-0.162414828	0.579066966	< 0.4	Negative	P >= 0.05
DNAI	IS	0.397802198	0.158950775	< 0.4	Positive	P >= 0.05
MI	IS	0.465788204	0.093231094	0.4 - 0.6	Positive	P >= 0.05
PMI	IS	-0.112087912	0.702833163	< 0.4	Negative	P >= 0.05
SAb	IS	-0.037362637	0.899093097	< 0.4	Negative	P >= 0.05
SM	KYN	-0.046153846	0.875502538	< 0.4	Negative	P >= 0.05
SD	KYN	-0.444444713	0.111339458	0.4 - 0.6	Negative	P >= 0.05
EV	KYN	0.362652287	0.202544581	< 0.4	Positive	P >= 0.05
DNAI	KYN	-0.068131868	0.81698388	< 0.4	Negative	P >= 0.05
MI	KYN	0.192054852	0.510684418	< 0.4	Positive	P >= 0.05
PMI	KYN	0.112087912	0.702833163	< 0.4	Positive	P >= 0.05
SAb	KYN	0.081318681	0.782274751	< 0.4	Positive	P >= 0.05
SM	KYNA	0.120879121	0.68060702	< 0.4	Positive	P >= 0.05
SD	KYNA	-0.074807526	0.799369821	< 0.4	Negative	P >= 0.05
EV	KYNA	0.700831107	0.005236544	>= 0.6	Positive	P < 0.05
DNAI	KYNA	0.046153846	0.875502538	< 0.4	Positive	P >= 0.05
MI	KYNA	0.125829041	0.668196445	< 0.4	Positive	P >= 0.05
PMI	KYNA	0.098901099	0.73658537	< 0.4	Positive	P >= 0.05
SAb	KYNA	-0.103296703	0.725282211	< 0.4	Negative	P >= 0.05
SM	NiA	0.081318681	0.782274751	< 0.4	Positive	P >= 0.05
SD	NiA	0.246424792	0.395723233	< 0.4	Positive	P >= 0.05
EV	NiA	0.300356189	0.2967661	< 0.4	Positive	P >= 0.05
DNAI	NiA	0.037362637	0.899093097	< 0.4	Positive	P >= 0.05
MI	NiA	-0.196469906	0.500824036	< 0.4	Negative	P >= 0.05
PMI	NiA	-0.120879121	0.68060702	< 0.4	Negative	P >= 0.05
SAb	NiA	-0.116483516	0.69169125	< 0.4	Negative	P >= 0.05
SM	Xa	0.265934066	0.3581134	< 0.4	Positive	P >= 0.05
SD	Xa	-0.088008854	0.764804391	< 0.4	Negative	P >= 0.05
EV	Xa	0.556215165	0.038870784	0.4 - 0.6	Positive	P < 0.05
DNAI	Xa	0.147252747	0.615418762	< 0.4	Positive	P >= 0.05
MI	Xa	0.130244095	0.657192879	< 0.4	Positive	P >= 0.05

PMI	Xa	0.415384615	0.139665948	0.4 - 0.6	Positive	P >= 0.05
SAb	Xa	-0.301098901	0.295514466	< 0.4	Negative	P >= 0.05
